# Nationwide Monitoring and Risk Assessment of Pesticide Residues in Fishery Products

**DOI:** 10.3390/toxics13090778

**Published:** 2025-09-14

**Authors:** Dong-ju Kim, Eun-been Oh, Jee-hyo Moon, Jeong-won Choi, Tae-hwa Kim, Seok-hee Lee, Ju-yeon Park, Chan-hyeok Kwon, Kee-sung Kyung

**Affiliations:** 1 Department of Environmental and Biological Chemistry, Chungbuk National University, Cheongju 28644, Republic of Korea; kimdj6746@naver.com (D.-j.K.); gsw06059@naver.com (E.-b.O.); myulyng14@gmail.com (J.-h.M.); ww8597@naver.com (J.-w.C.); 2 Analysis Technology and Tomorrow, Daegu 42703, Republic of Korea; thkim@atnt.co.kr; 3 Department of Food Science and Biotechnology, Dongguk University, Goyang 10326, Republic of Korea; seokhee@dongguk.edu; 4 Pesticide and Veterinary Drugs Residues Division, National Institute of Food and Drug Safety Evaluation, Ministry of Food and Drug Safety, Osong 28159, Republic of Korea; jy520@korea.kr (J.-y.P.); chkwon@korea.kr (C.-h.K.)

**Keywords:** fishery products, monitoring, multi-residue, pesticide, risk assessment

## Abstract

Global production of fisheries and aquaculture products continues to increase, with the fisheries sector increasingly considered essential for global food security and nutrition. As public demand for seafood increases, implementation of safety management to minimize risks and ensure the safety of seafood products becomes important. This study was conducted to monitor 198 chemicals, comprising 161 pesticides and 37 pesticide metabolites, and to assess their risks in saltwater and freshwater fish in the Republic of Korea, based on the analysis of 471 fishery samples (298 saltwater and 173 freshwater). Among the fifteen saltwater fish species analyzed, pesticides were detected in eight species (croaker, flatfish, sea bass, flounder, snapper, yellow tail, salmon, and gizzard shad) and in all seven freshwater species (carp, catfish, crucian carp, eel, leather carp, loach, and trout). Four pesticides (ethoxyquin, lufenuron, metaflumizone, and propiconazole) were detected in saltwater fish, while nine pesticides (ethoxyquin, ipfencarbazone, isoprothiolane, lufenuron, metaflumizone, oxadiargyl, pendimethalin, phoxim, and trichlorfon) were found in freshwater fish. Ethoxyquin was the most frequently detected pesticide in both fish types, mostly in the form of its metabolite, the ethoxyquin dimer. The estimated daily intake (EDI) was calculated based on the maximum concentrations of pesticides detected and the average consumption of fishery products by sex and age group. The hazard quotients, expressed as a percentage of acceptable daily intake (%ADI) and calculated using the EDI and ADI of the pesticides detected, were evaluated to be no more than 5.6%. These results suggest that consumption of saltwater and freshwater fish in the Republic of Korea poses a low risk to human health. This approach can be applied to pesticide residue monitoring and risk assessment in the fisheries sector, providing valuable data for evaluating contamination levels and supporting the regulation and management of chemical residues.

## 1. Introduction

The production of fisheries and aquaculture products continues to grow globally, and the fisheries sector has increasingly been recognized in the 21st century as essential for global food security and nutrition [1]. According to the Food and Agriculture Organization of the United Nations (UN-FAO) and its report, ‘The State of World Fisheries and Aquaculture’, global average annual per capita consumption of fishery products has increased from 9.9 kg in the 1960s to 20.2 kg in 2013–2015, with an average annual growth rate of 3.2% over the past 50 years [1]. According to the Ministry of Oceans and Fisheries, the Republic of Korea ranked first among major countries such as China, the European Union (EU), Japan, and the United States of America (USA) in seafood consumption in 2022, with an annual per capita consumption of 68.4 kg [2]. The increase in seafood consumption can be attributed to improved national income levels and growing societal interest in health, which have led to increased consumer preference for seafood as a low-fat and high-protein food [3].

The Republic of Korea’s seafood self-sufficiency rate decreased from 84.6% in 2011 to 71.4% in 2016 and has since remained in the range of 65.5 to 68.9% until 2020. Meanwhile, according to seafood trade statistics, the total value of imports increased from USD 4.19 billion in 2011 to USD 6.96 billion in 2022 [2]. This indicates that the Republic of Korea’s seafood supply is increasingly more dependent on imports than on domestic production. As public demand for seafood remains high, the implementation of safety management to minimize risks and ensure the safety of seafood products is essential [4].

In the Republic of Korea, residues of ethoxyquin and ethoxyquin dimers are consistently detected in fishery products, and the maximum residue limit (MRL) for ethoxyquin, which is defined as the sum of ethoxyquin and ethoxyquin dimers, is set at 1.0 mg/kg for fish and 0.2 mg/kg for crustaceans [5]. Several countries, including Australia [6], the European Union [7], Japan [8], and the USA [9], have established MRLs to regulate pesticide residues in both domestically produced and imported fish and food products. Codex sets extraneous MRLs (EMRLs) based on monitoring data for persistent pesticide components that remain as environmental contaminants in food [10]. Among the MRLs established by Codex for diadromous fish, saltwater fish and trout, only lindane currently has a specified MRL for regulatory management. The United States Food and Drug Administration (US-FDA) conducts annual monitoring of agricultural commodities, livestock, and fish to regulate pesticide residues [11].

The process of bioconcentration in aquatic organisms can be divided into two pathways of absorption by which residual substances are introduced into the aquatic environment through rainfall and the consumption of contaminated food [12]. Seafood hazards can be categorized into chemical factors, such as pesticides, heavy metals, radiation, and veterinary drugs, and biological factors, including Vibrio bacteria, norovirus, parasites, and marine toxins [13]. Pesticide residues in seafood differ from those in agricultural products because they typically result from indirect exposure rather than direct application. Instead, these residues occur through indirect factors such as pesticide runoff during rainfall, leaching into groundwater, or discharge into rivers, all of which contaminate water resources [14]. Hence, water quality in aquaculture is a key factor in determining the health of fish [15]. However, in practice, factors such as high-density breeding, pollution, and eutrophication of water bodies often lead to a decline in water quality and outbreaks of fish diseases [16]. Therefore, antibiotics are used globally as aquaculture drugs or feed additives to control fish diseases [17]. Additionally, a fungicide, ethoxyquin, is widely used as an antioxidant in animal feed and an ignition and exploitation inhibitor during marine transportation. Compounds with high environmental persistence and a high log K_ow_ tend to bioaccumulate in fish bodies. This means that pesticides used as antibiotics and feed additives in aquaculture can contaminate fish farms [18]. Therefore, risk assessments that monitor pesticide residues potentially remaining in seafood are necessary to ensure the safety of seafood [19].

This study was conducted to validate a multi-residue analysis method for fishery products suggested by the Korean Ministry of Food and Drug Safety (MFDS) using liquid chromatography with tandem mass spectrometry (LC-MS/MS) to simultaneously detect 198 chemicals, including 161 pesticides. In addition, pesticide residues in saltwater and freshwater fish were monitored over a two-year period, and a risk assessment was conducted based on various consumption patterns and different age groups of the detected samples [20].

Recent studies have also reported multiresidue monitoring and risk assessment of pesticides in diverse fishery products and seafood commodities, highlighting the necessity of continuous surveillance and methodological improvements [21,22]. This approach can be applied in monitoring and risk assessment to provide valuable data for evaluating contamination levels in fishery products, as well as for the regulation and management of pesticide residues.

## 2. Materials and Methods

### 2.1. Reagents and Instruments

Acetone, acetonitrile, methanol, and water of high-performance liquid chromatography (HPLC) grade were purchased from Honeywell (Charlotte, NC, USA). Formic acid (99.0%) was purchased from Samchun (Seoul, Republic of Korea). Ammonium formate (95.0%) and dimethyl sulfoxide (99.0%) were purchased from Kanto Chemical (Tokyo, Japan). The QuEChERS original method pouch (4 g MgSO_4_, 1 g NaCl) and the dispersive solid-phase (d-SPE) clean-up sorbent tubes (150 mg MgSO_4_, 25 mg PSA, 25 mg C_18_) were purchased from Taesan Science (Gunpo, Republic of Korea) and Chiral Technology Korea (Daejeon, Republic of Korea), respectively. Polytetrafluoroethylene filters were purchased from GVS Filter Technology (Morecambe, UK). The extractor used was the 2010 Geno/Grinder^®^ from SPEX SamplePrep LLC (Metuchen, NJ, USA), and the centrifuge used was the Combi-514R and Smart-R17 from Hanil Scientific Industrial (Gimpo, Republic of Korea).

### 2.2. Chemicals

All standards for the 198 chemicals, including 161 pesticides, used in the analysis were purchased from AccuStandard (New Haven, CT, USA), Chem Service (West Chester, PA, USA), HPC Standards GmbH (Borsdorf, Germany), Kemidas (Gunpo, Republic of Korea), LGC Standards (Teddington, UK), Sigma-Aldrich (St. Louis, MO, USA), and Wako Pure Chemical (Osaka, Japan). Each standard was diluted with acetone, acetonitrile, methanol, and water based on its solubility in the respective solvents. Individual stock solutions were prepared for each standard, and mixed standard solutions were then prepared for LC-MS/MS analysis. All solutions were stored at −20 °C until use.

### 2.3. Sample Selection and Collection

#### 2.3.1. Background of Sample Selection

Fish species for pesticide residue analysis in seafood were classified into saltwater and freshwater fish based on their habitat and were selected based on food consumption data reported by the Korea Health Industry Development Institute (KHIDI) [23]. A total of 22 species were selected, prioritizing those with high consumption [23], domestic pesticide residue detection records [24,25,26], and species with seasonally high consumption. The seafood samples selected for each species are presented in Table 1. In addition, sample collection sites were selected from nine locations nationwide (Busan, Daegu/Ulsan, Gangwon, Chungcheong/Daejeon/Sejong, Gyeonggi/Incheon, Gyeongsang, Jeju, Jeolla/Gwangju, and Seoul) based on the population size and density of the administrative districts reported by the Republic of Korea. Considering that the regional seafood distribution system primarily consists of agricultural and fishery wholesale markets and large-scale supermarkets, samples were collected from these locations at least monthly and quarterly during a two-year period (2023–2024).

#### 2.3.2. Sample Collection

**Figure 1:**
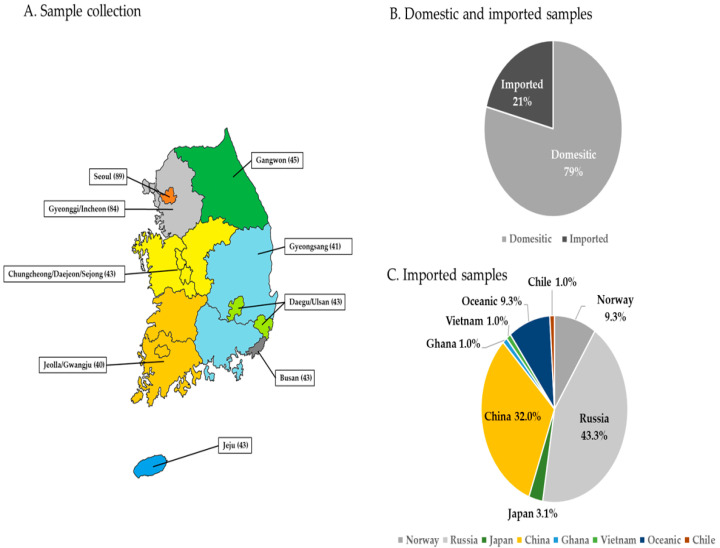
Map of sample collection regions (**A**) and percentage of domestic and imported samples among all samples (**B**,**C**). Figures in parentheses indicate the number of samples collected.

A total of 471 seafood samples were collected over a period of two years (2023–2024) from markets in the Republic of Korea, including 298 saltwater and 173 freshwater fish samples. A total of 115 out of the 471 samples were collected in 2023, and the remaining 356 samples were collected in 2024. Among the collected samples, 374 samples were domestically produced and 97 samples were imported, representing a ratio of 79:21. Sample collection distribution by region, domestic and imported products ratio, and imported products collection status are presented in Figure 1. Samples were collected using disposable gloves as a mandatory practice to minimize changes in quality and composition. To prevent contamination, each fish was individually packaged and transported on the day of collection. The quantity and analytical parts of each sample were determined based on MFDS guidelines [26] to ensure representativeness in residue analysis, and samples were acquired in amounts of at least 1 kg. Each collected sample was used as an analytical sample by isolating the muscle parts, including the skin, with the head, tail, bones, intestine, and scales removed. For small-sized fish such as loaches, the entire body was used. Subsequently, the analytical parts of the samples were separated in the laboratory, frozen, and homogenized on dry ice. The samples were then stored at −20 °C until analysis.

### 2.4. Analysis

In this study, pesticide residues in the samples were analyzed using a multi-class pesticide multi-residue method based on the QuEChERS pretreatment method proposed by MFDS [27]. For saltwater and freshwater fish samples, 5 g of each sample was mixed with 10 mL of acetonitrile and shaken at 1300 rpm for 1 min. Then, 4 g of MgSO_4_ and 1 g of NaCl were added, followed by shaking at 1300 rpm for 10 min. The mixture was then centrifuged at 1790× *g* and 4 °C for 10 min using a Combi-514R centrifuge (Hanil Scientific Industrial, Gimpo, Republic of Korea, S750-4B rotor). Subsequently, 1 mL of the supernatant was transferred to a 2 mL centrifuge tube containing 150 mg of MgSO_4_, 25 mg of C_18_, and 25 mg of primary-secondary amine (PSA). The mixture was shaken for 1 min and then centrifuged at 9360× *g* and 4 °C for 2 min using a Smart-R17 centrifuge (Hanil Scientific Industrial, Gimpo, Republic of Korea, A1.5M-24 rotor). The resulting supernatant was filtered through a 0.2 μm polytetrafluoroethylene membrane filter and used as the test solution. To validate the multi-class pesticide multi-residue method for pesticide residues in seafood, a representative matrix was selected based on species with high intake from both saltwater and freshwater fish. Flat fish and eel were selected as representative matrices for saltwater and freshwater fish, respectively, and were used for validation. Additionally, homogeneous samples from each representative matrix, in which none of the 198 chemicals, including 161 pesticides, were detected, were used as control matrices for matrix-matched calibration.

### 2.5. Method Validation

The analytical instrument used was LC-MS/MS, and its operating conditions are summarized in Table 2. Additionally, the multiple-reaction monitoring (MRM) conditions for 198 chemicals, including 161 pesticides, are detailed in Table S1. The multi-class pesticide multi-residue method for seafood was validated for linearity, limit of detection (LOD), limit of quantification (LOQ), recovery, and repeatability following the Codex Guidelines (CAC/GL 40) in Table S2.

Matrix-matched calibration was employed to minimize the effects of the sample matrix. Representative matrix samples of saltwater and freshwater fish were pre-treated using the method described in Section 2.4 and used to prepare standard solutions for the construction of calibration curves. The matrix correction rate was set to 90%. Calibration curves were constructed using peak areas over a concentration range of 0.002–0.1 mg/L for both saltwater and freshwater fish. The linearity of the calibration curve was expressed as the coefficient of determination (R^2^). The slopes of the calibration curves for the test chemicals prepared in pure solvent were compared with the slopes of the calibration curves matched with each representative matrix to determine and calculate the matrix effect (ME) using Equation (1). The recovery test to evaluate the suitability of the analytical method was repeated five times after spiking the samples with mixed standard solutions at 1×, 2×, or 10× LOQ levels. Subsequently, the mean recovery and coefficient of variation (CV) were calculated to evaluate the accuracy, precision, and reproducibility of this method.

Matrix effect (%) = (Slope of calibration of matrix/slope of calibration in solvent − 1) × 100(1)

### 2.6. Risk Assessment

Daily intake of the monitored chemicals was investigated by the MFDS [28], Rural Development Administration (RDA) [29], European Food Safety Authority (EFSA) [30], Environmental Protection Agency (EPA) [31], World Health Organization (WHO) [32] and Food Safety Commission of Japan (FSCJ) [33]. Using the food intake obtained from the 8th KHIDI (2019–2021), and the weights in Table 3, the groups were divided into 1–2, 3–6, 7–12, 13–19, 20–64, ≥65, ≤20, and ≥20 years old, and male and female groups. The daily intakes of fishery products were investigated based on the average intake by sex and age for all consumers.

The percentage of acceptable daily intake (%ADI) was calculated by combining the estimated daily intake (EDI) with the reference acceptable daily intake (ADI, mg/kg BW/day). The calculation considered the average body weight (BW) by age and sex to account for population differences. Equations (2) and (3) were applied for the risk assessment [34], and the reference ADI values for each pesticide and the sources of the ADI values have been provided in Supplementary Table S6.

(2) $$\begin{array}{c} \mathrm{EDI}\;(\mathrm{mg}/\mathrm{kg}\;\mathrm{BW}/\mathrm{day})=\\ \mathrm{Detected}\;\mathrm{pesticide}\;\mathrm{concentration}\;(\mathrm{mg}/\mathrm{kg})\;\times \;\mathrm{food}\;\mathrm{intake}\;(\mathrm{kg}/\mathrm{day})/\mathrm{BW}\;(\mathrm{kg}) \end{array}$$

%ADI = EDI (mg/kg BW/day)/ADI (mg/kg BW/day) × 100(3)

## 3. Results and Discussion

### 3.1. Method Validation

Liquid chromatography–electrospray ionization-mass spectrometry (LC-ESI-MS) enables the analysis of a wide range of highly sensitive compounds [35]; however, matrix components co-eluting with a target analyte in a sample can affect the ionization process, causing ion suppression or enhancement [36]. This effect is termed the matrix effect and can lead to issues such as reduced precision and accuracy [37]. The matrix effect was classified into three levels: weak, medium, and strong. The calculated ME values were categorized as weak (−20% to 20%), intermediate (−50% to −20% or 20% to 50%), and strong (<−50% or >50%). In cases where matrix effects were intermediate or strong, applying appropriate correction methods was considered essential, as such effects may significantly influence analytical accuracy [38].

**Figure 2:**
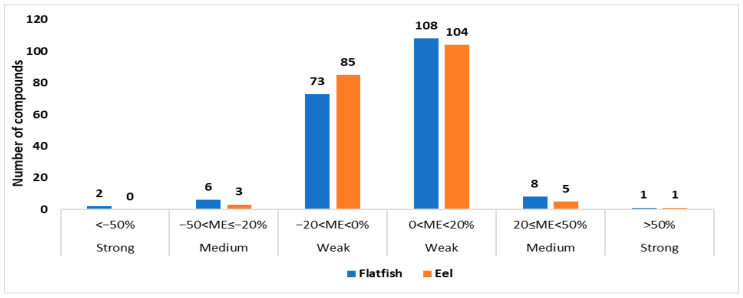
Matrix effect distribution of flatfish and eel, representative species of saltwater fish and freshwater fish.

To evaluate the matrix effect on the analytes under LC-MS/MS-ESI conditions, the slope of the calibration curve for the mixed standard solution diluted with pure acetonitrile was compared with the slope of each analyte component in the standard solution matrix-matched with representative samples (flatfish and eel). Subsequently, the matrix effects of each analyte component were calculated. Among the target analytes, 181 chemicals (91.4%) in flatfish were within the weak range, 14 chemicals (7.0%) within the medium range, and the remaining 3 chemicals (1.5%) within the strong range. In eels, 189 chemicals (95.5%) were within the weak range, 8 chemicals (4.0%) within the medium range, and 1 chemical (0.5%) within the strong range, as shown in Figure 2 and Table S3. The results of the matrix effect analysis showed that most of the chemicals were within the weak range. However, 17 components in flatfish and 9 components in eel were classified within the medium and strong ranges, indicating that calibration of the matrix effect is necessary to perform an accurate quantitative analysis. In this study, to minimize the impact of matrix effects on the analysis of target chemicals in seafood and to reduce errors caused by the matrix, the matrix-matched method, the most common approach to prevent matrix effects in LC-MS/MS, was selected [39]. This method can be used to accurately quantify target components in samples [40].

**Figure 3:**
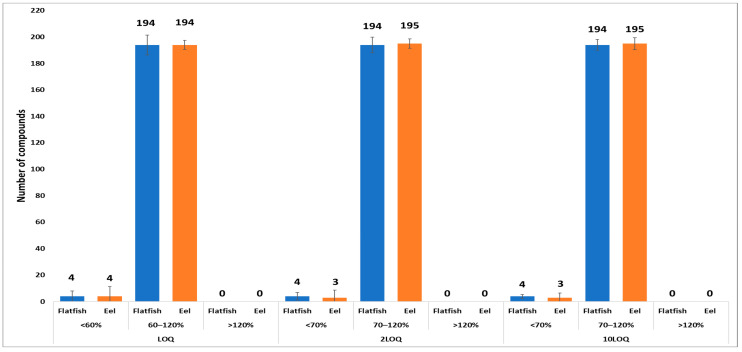
Comparison of the recoveries for flatfish (saltwater fish) and eel (freshwater fish) as representative samples.

Subsequently, residue analysis was performed to demonstrate the applicability of a multi-class pesticide multi-residue method for a total of 198 chemicals, including 161 pesticides, in seafood. The results showed that the linearity of the matrix-matched calibration curves for both saltwater and freshwater fish was within the range of 0.002–0.1 mg/L, with all R^2^ values greater than or equal to 0.98, meeting the validation criteria required by Codex guidelines (CAC/GL 40). The chemicals that could be analyzed using the multi-class pesticide multi-residue method for pesticides included a total of 194 chemicals in flatfish and 194 chemicals in eel, as they satisfied all the recovery ranges and precision required by Codex guidelines (CAC/GL 40). The average recoveries for flatfish and eel were 62.5–117.1 and 62.6–116.5%, respectively, as illustrated in Figure 3 and Table S4. Specifically, at the LOQ level, recoveries were within 60–120% with standard deviations below 30%, whereas at the 2LOQ and 10LOQ levels, recoveries were within 70–120% with standard deviations below 20%. The components that could not be quantitatively analyzed using the analysis method for pesticides in seafood were simazine-2-hydroxy, terbuthylazine-2-hydroxy, and terbuthylazine-desethyl-2-hydroxy in both saltwater and freshwater fish. Additionally, penoxsulam was not applicable to saltwater fish, and acynonapyr is not applicable to freshwater fish. The low recoveries of pesticides when using the QuEChERS sample preparation method can be attributed to factors such as the degradation of pesticides during the pretreatment process or instrumental analysis, insufficient partitioning into the acetonitrile layer, or adsorption onto sorbents during the cleanup process [41].

The analytical method validated in this study demonstrated that 194 of 198 chemicals, including 161 pesticides, in saltwater fish (flatfish) and freshwater fish (eel) satisfied the international standards for recovery and standard deviation at all test levels. Based on this validation, the residual amounts of seafood samples distributed in the Republic of Korea were investigated.

### 3.2. Detection Rate in Fishery Products

**Figure 4:**
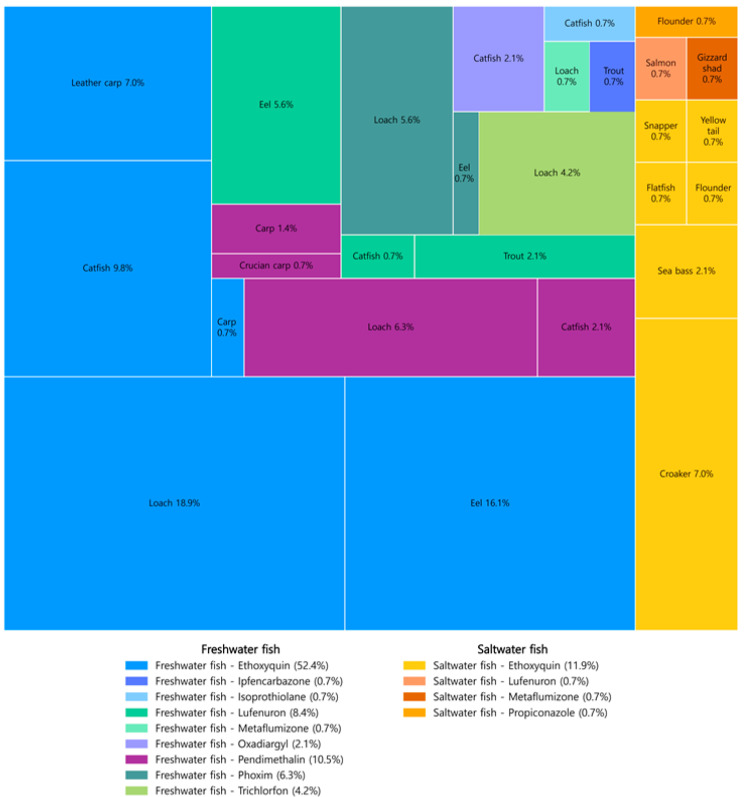
Detection rates of pesticides by fish species in saltwater and freshwater.

The monitoring results for 298 saltwater fish samples and 173 freshwater fish samples purchased during 2023–2024 are presented in Table 4 and Figure 4. The detection rates of the target compounds in saltwater and freshwater fishes were 6.7 and 56.6%, respectively. Among the detected species, croaker accounted for 50% of the saltwater fish, whereas loach represented 36.7% of the freshwater fish, showing the highest detection in each fish. The target chemicals detected in saltwater fish were ethoxyquin, ethoxyquin dimer, lufenuron, metaflumizone, and propiconazole, whereas those in freshwater fish were ethoxyquin, ethoxyquin dimer, lufenuron, trichlorfon, pendimethalin, metaflumizone, phoxim, ipfencarbazone, oxadiargyl, and isoprothiolane. Ethoxyquin, ethoxyquin dimer, lufenuron, and metaflumizone were detected in both saltwater and freshwater fish. The log K_ow_ values of the detected chemicals were all greater than 3, indicating a high potential for bioaccumulation. The report on the feeding of ethoxyquin-containing feed indicated that ethoxyquin and ethoxyquin metabolites were detected in feed-fed fish [42]. Ethoxyquin was mainly detected as an ethoxyquin dimer; however, the demethylation-rearomatization product (2,4-dimethyl-6-ethoxyquinoline, DMEQ), deethylation product hydroxyquin (1,2-dihydro-6-hydroxy-2,2,4-trimethylquinoline, DEQ), and its corresponding quinone imine (2,6-dihydro-2,2,4-trimethyl-6-quinolone, QI) were also detected, although often at negligible intensities [43]. Ethoxyquin dimer was detected in 17 saltwater fish samples and 67 freshwater fish samples, whereas ethoxyquin was detected in 2 saltwater fish samples from croaker and 1 freshwater fish sample from loach. After feeding salmon an ethoxyquin-containing feed for 90 days, followed by a 90-day depuration period, the half-life of ethoxyquin was determined to be 7.8 days, while that of ethoxyquin dimer was 71 days [44]. Furthermore, after feeding salmon with an ethoxyquin-containing feed for 12 days, the residual amount of ethoxyquin dimers comprised 98% of the total ethoxyquin and ethoxyquin dimers, suggesting that ethoxyquin transferred from the feed remained in the fish in its dimeric form rather than the original form during aquaculture [45]. Therefore, this study was also consistent with 3% and 97% detection rates of ethoxyquin and its dimer [45]. According to the international residue definition, ethoxyquin is expressed as the sum of the parent compound and its dimer. In this study, the results were reported based on this residue definition, meaning that the values represent the combined residues of ethoxyquin and ethoxyquin dimer.

### 3.3. Residue in Fishery Products

The concentration of ethoxyquin in saltwater fish ranged between 0.01 and 0.97 mg/kg, with a mean residual concentration of 0.22 mg/kg and the highest concentration observed in croaker (0.97 mg/kg), whereas those in sea bass, flatfish, flounder, snapper, and yellow tail were all less than 0.04 mg/kg. The residue of ethoxyquin in freshwater fish ranged between 0.01 and 0.45 mg/kg with a mean residual concentration of 0.07 mg/kg and the highest concentration observed in eels (0.45 mg/kg), while those in loach, catfish, leather carp, and carp were less than 0.15 mg/kg. The residual amount of ethoxyquin detected in both saltwater fish and freshwater fish was less than its MRL of 1.0 mg/kg set by the MFDS. Choi et al. [46] reported that the concentration of ethoxyquin in farmed fish ranged between 0.009 and 0.024 mg/kg (flounder) in saltwater fish and 0.005–0.309 mg/kg in freshwater fish (loach, eel, and catfish). Additionally, ethoxyquin dimers have been detected in saltwater fish such as croaker, sea bass, flatfish, flounder, snapper, and yellow tail ranging from 0.001 to 1.790 mg/kg and in freshwater fish such as loach, eel, catfish, leather, crusian carp, and carp, ranging from 0.002 to 2.828 mg/kg, similar to the fish species detected in this study. Several pesticides, including pendimethalin, phoxim, trichlorfon, oxadiargyl, ipfencarbazone, and isoprothiolane, were detected only in freshwater fish. Propiconazole was found in saltwater fish, while lufenuron and metaflumizone were present in both saltwater and freshwater fish. The herbicide pendimethalin and the fungicide isoprothiolane were detected in freshwater fish. These findings suggest that the pesticides applied during the rice cultivation period were transported and remained in aquaculture pond soils due to repeated irrigation and drainage, as well as heavy-rainfall-induced runoff [47,48]. Trichlorfon is a veterinary drug used to treat parasitic diseases in fish, and it was detected in loach because it is used in farmed fish [49]. Even if trichlorfon was metabolized to dichlorvos, since dichlorvos is included in the target compounds, we believe it would have been detected if it was present above its LOQ. Additionally, because the ADI of trichlorfon was very low (0.002 mg/kg), it may pose a potential risk to humans. Therefore, continuous monitoring is necessary to assess the safety of saltwater and freshwater fish.

### 3.4. Risk Assessment by Gender and Age for Average Consumers

When a total of 194 chemicals, including pesticides, veterinary drugs, and some of their metabolites, were monitored in a total of 471 fish samples, 10 chemicals were detected in 118 of the samples. Potential risk assessment was conducted based on the concentrations of pesticides detected in the samples. However, there was a possibility of underestimating the exposure for the entire population. To complement this, a detailed risk assessment was performed by age and gender, including vulnerable groups, such as children and the elderly. The results of this assessment are presented in Table 5 and Table S5.

Seven of the ten detected pesticides, excluding ethoxyquin, phoxim, and trichlorfon, showed an ADI of less than 1% for all average consumers. In the case of ethoxyquin, the %ADI was less than 1% for all samples, except croaker. Additionally, there were no significant differences in food intake between sexes across all detected samples. In croaker, ethoxyquin showed the highest %ADI at 4.3% for the 1–2-year age group. This is because although the intake for the 1–2-year group (2.8 g) and the overall group (2.0 g) showed minimal differences, the average body weight for the 1–2-year group (12.6 kg) was approximately five times lower than that of the overall group (59.7 kg). Consequently, the %ADI for the 1–2-year group was relatively higher. Body weight is regarded as a key exposure factor in risk assessment because the concentration of a contaminant in the body can vary depending on body weight, even when the same amount of contaminant is ingested. Therefore, exposure to contaminants can differ depending on the body weight [50]. For phoxim, the highest %ADI (1.2%) was observed in eel for the more than 65-year group, whereas for trichlorfon, the highest %ADI (0.6%) was detected in loach for the same age group. This is likely because the consumption of loaches and eels increases with age, resulting in higher risk assessment in older age groups.

According to the MFDS, a risk assessment is considered hazardous if it exceeds 100%, whereas values not more than 100% are deemed safe. Previous studies conducted in the Republic of Korea also detected pesticides such as DDT, isoprothiolane, oxadiazon, pendimethalin, and thifluzamide in seafood; however, the %ADI values were reported to be not more than 1.07%, indicating a low level of risk [51]. Similarly, in this study, although pesticides were detected in seafood, the risk was assessed to be very low considering seafood consumption in the Korean population.

## 4. Conclusions

This study was conducted to validate the multi-class pesticide multi-residue method for the analysis of 198 chemicals, including 161 pesticides, in fishery products suggested by the MFDS, to monitor those residues, and to perform a risk assessment on them. Fishery product sampling was conducted by selecting saltwater and freshwater fish samples, considering factors such as intake rate, domestic pesticide detection history, and country of origin. A total of 471 samples were collected, encompassing 298 samples of 15 saltwater fish species and 173 samples of 7 freshwater fish species, from across the country. As a result of monitoring, four chemicals were detected 20 times in eight saltwater fishes, while nine chemicals were detected 98 times in seven freshwater fishes. The most frequently detected fish species were croaker in saltwater fish and loach in freshwater fish. The detection rates of the chemicals were 6.7% in saltwater fish and 56.6% in freshwater fish, with ethoxyquin being the most frequently detected chemical. The concentrations of chemicals detected in saltwater and freshwater fish ranged from 0.01 to 1.88 mg/kg. The chemicals detected in fishery products are presumed to originate from contaminated water and soil, primarily due to agricultural practices, as well as from the feed used in aquaculture during breeding of fish. The risk assessment of the fishery products indicated that the %ADI was less than 5.6% for all average consumers, suggesting that consuming saltwater and freshwater fish may be safe. Furthermore, as residues of chemicals tend to decrease during the washing and cooking processes, the actual exposure risk for consumers is expected to be lower. Nevertheless, because there are pesticides like trichlorfon with a very low ADI of 0.002 mg/kg, continuous monitoring for safety assessment is considered necessary. In addition, demographic factors such as age- and gender-specific consumption patterns, differences in toxicokinetics, and body-weight adjustments may influence exposure estimates. Therefore, we believe that considering these variables in future risk assessments will enable a more comprehensive evaluation of consumer safety.

## Figures and Tables

**Table 1:** The fishery products selected based on habitat-based fish classification.

Classification	Fishery Product
Saltwater fish	Convict grouper ^2^, croaker ^1^, filefish ^2^, flatfish ^1^, flounder ^2^, gizzard shad ^2^, mackerel ^1^, mullet ^2^, pollock ^1^, rockfish ^2^, salmon ^2^, sea bass ^2^, snapper ^2^, tuna ^2^, yellow tail ^2^
Freshwater fish	Catfish ^1^, carp ^2^, crucian carp ^2^, eel ^1^, leather carp ^2^, loach ^1^, trout ^2^

^1^ Fishery products with high consumption based on the Korea Health Industry Development Institute [23], ^2^ Fishery products with domestic detection records and seasonally high consumption.

**Table 2:** Instrumental conditions of LC-MS/MS.

Instrument	Nexera X3, Shimadzu, Japan		
Data processing	Labsolution (version 5.98)		
Column	Phenomenex Kinetex C_18_ (150 mm L.× 2.1 mm I.D., 2.6 μm particle size)		
Flow rate	0.4 mL/min		
Detector	Triple-quadruple spectrometer, LCMS-8050, Shimadzu, Japan		
Mobile phase	A: 1 mM ammonium formate with 0.1% formic acid in water B: 1 mM ammonium formate with 0.1% formic acid in methanol		
	Time (min)	A (%)	B (%)
	1.00	90	10
	3.00	45	55
	10.50	0	100
	12.00	0	100
	12.01	90	10
	15.00	90	10
Injection volume	5 µL		
Ionization source	Electrospray ionization (ESI)		
Polarity	Positive and negative		
Interface temperature	150 °C		
Nebulizing gas flow	3.0 L/min		
Drying gas flow	10.0 L/min		
Heating gas flow	10.0 L/min		
DL temperature	250 °C		
Heat block temperature	400 °C		

**Table 3:** Body weights by gender and age group.

Category	Average	Age								Gender	
		1–2	3–6	7–12	13–19	20–64	65 Older	20 Under	20 Older	Male	Female
Average body weight (kg BW)	59.71	12.60	19.61	38.40	61.66	65.96	60.39	40.01	64.27	65.60	54.99

**Table 4:** Concentration of pesticides detected in fishery products.

Compound	Species of Fish		Sample Size	Detection Number			Detection Rate (%)	Concentration (mg/kg)		MRL ^c^ (mg/kg)
				Total	Domestic	Imported		Range ^a^	Mean ^b^	
Ethoxyquin	Saltwater fish	Croaker	44	10	2	8	22.7	0.06–0.97	0.37	Korea: 1.0 (Fish)
		Flatfish	44	1	1	-	2.3	0.01	0.01	
		Flounder	13	1	1	-	7.7	0.02	0.02	
		Sea bass	12	3	1	2	25.0	0.01–0.02	0.01	
		Snapper	8	1	-	1	12.5	0.03	0.03	
		Yellow tail	8	1	1	-	12.5	0.04	0.04	
	Freshwater fish	Carp	8	1	-	1	12.5	0.03	0.03	
		Catfish	44	14	14	-	31.8	0.01–0.04	0.02	
		Eel	44	23	23	-	52.3	0.02–0.45	0.12	
		Leather carp	11	10	3	7	90.9	0.05–0.11	0.08	
		Loach	44	27	19	8	61.4	0.01–0.14	0.06	
Ipfencarbazone	Freshwater fish	Trout	11	1	1	-	9.1	0.01	0.01	Japan: 0.04 (Seafood)
Isoprothiolane	Freshwater fish	Catfish	44	1	1	-	2.3	0.01	0.01	Japan: 3.0 (Seafood)
Lufenuron	Saltwater fish	Salmon	11	1	-	1	9.1	0.04	0.04	-
	Freshwater fish	Catfish	44	1	1	-	2.3	0.01	0.01	
		Eel	44	8	8	-	18.2	0.01–1.58	0.36	
		Trout	11	3	3	-	27.3	0.02	0.02	
Metaflumizone	Saltwater fish	Gizzard shad	11	1	1	-	9.1	0.03	0.03	-
	Freshwater fish	Loach	44	1	1	-	2.3	0.01	0.01	
Oxadiargyl	Freshwater fish	Catfish	44	3	3	-	6.8	0.02–0.04	0.03	Japan: 0.02 (Seafood)
Pendimethalin	Freshwater fish	Carp	8	2	2	-	25.0	0.01	0.01	Japan: 0.03 (Seafood)
		Catfish	44	3	3	-	6.8	0.01–0.02	0.01	
		Crucian carp	11	1	1	-	9.1	0.03	0.03	
		Loach	44	9	8	1	20.5	0.01–0.93	0.20	
Phoxim	Freshwater fish	Eel	44	1	1	-	2.3	0.02	0.02	-
		Loach	44	8	5	3	18.2	0.03–1.88	0.58	
Propiconazole	Saltwater fish	Flounder	13	1	1	-	7.7	0.01	0.01	-
Trichlorfon	Freshwater fish	Loach	44	6	5	1	13.6	0.03–0.42	0.16	Japan: 0.01 (Eel)

^a^ The single figure is represented as the mean value because the standard deviation value is almost 0, ^b^ Average of 3 replicates, ^c^ Maximum residue limit.

**Table 5:** Assessment of dietary exposure to the target chemicals for the average consumption.

Chemical	Fishery Product	%ADI (Acceptable Daily Intake)										
		All Age	1–2	3–6	7–12	13–19	20–64	65 Older	20 Under	20 Older	Male	Female
Ethoxyquin	Carp	0.0 ^a^	- ^b^	-	-	-	0.0	0.0	-	0.0	0.0	0.0
	Catfish	0.0	-	-	-	-	0.0	0.0	-	0.0	0.0	0.0
	Croaker	0.6	4.3	3.4	0.5	0.5	0.5	0.7	0.9	0.6	0.7	0.6
	Eel	0.2	-	0.0	0.1	0.1	0.2	0.3	0.1	0.2	0.2	0.2
	Flatfish	0.0	-	0.0	0.0	0.0	0.0	0.0	0.0	0.0	0.0	0.0
	Flounder	0.0	-	-	-	-	0.0	0.0	-	0.0	0.0	0.0
	Leather carp	0.0	-	-	-	-	0.0	0.0	-	0.0	-	0.0
	Loach	0.0	-	0.0	0.0	0.0	0.0	0.1	0.0	0.0	0.0	0.0
	Sea bass	0.0	-	-	-	-	0.0	0.0	-	0.0	0.0	0.0
	Snapper	0.0	0.0	0.0	0.0	-	0.0	0.0	0.0	0.0	0.0	0.0
	Yellow tail	0.0	-	0.0	0.0	0.0	0.0	0.0	0.0	0.0	0.0	0.0
Ipfencarbazone	Trout	0.0	-	-	-	-	0.0	0.0	-	0.0	0.0	-
Isoprothiolane	Catfish	0.0	-	-	-	-	0.0	0.0	-	0.0	0.0	0.0
Lufenuron	Catfish	0.0	-	-	-	-	0.0	0.0	-	0.0	0.0	0.0
	Eel	0.2	-	0.0	0.1	0.1	0.2	0.3	0.1	0.2	0.2	0.2
	Salmon	0.0	0.0	0.0	0.0	0.0	0.0	0.0	0.0	0.0	0.0	0.0
	Trout	0.0	-	-	-	-	0.0	0.0	-	0.0	0.0	-
Metaflumizone	Gizzard shad	0.0	-	-	-	0.0	0.0	0.0	0.0	0.0	0.0	0.0
	Loach	0.0	-	0.0	0.0	0.0	0.0	0.0	0.0	0.0	0.0	0.0
Oxadiagyl	Catfish	0.0	-	-	-	-	0.0	0.0	-	0.0	0.0	0.0
Pendimethalin	Carp	0.0	-	-	-	-	0.0	0.0	-	0.0	0.0	0.0
	Catfish	0.0	-	-	-	-	0.0	0.0	-	0.0	0.0	0.0
	Crucian carp	0.0	-	-	-	-	0.0	-	-	0.0	-	0.0
	Loach	0.0	-	0.0	0.0	0.0	0.0	0.0	0.0	0.0	0.0	0.0
Phoxim	Eel	0.0	-	0.0	0.0	0.0	0.0	0.0	0.0	0.0	0.0	0.0
	Loach	0.7	-	0.1	0.2	0.1	0.6	1.2	0.1	0.8	0.8	0.6
Propiconazole	Flounder	0.0	-	-	-	-	0.0	0.0	-	0.0	0.0	0.0
Trichlorfon	Loach	0.3	-	0.0	0.1	0.1	0.3	0.6	0.1	0.4	0.4	0.3

^a^ Values shown as 0.0 were rounded to one decimal place; detailed unrounded data are provided in Supplementary Table S5, ^b^ Intake data was unavailable, so %ADI could not be calculated.

## Data Availability

The data presented in this study are available upon request from the corresponding author due to legal restrictions.

## Supplementary Materials

The following supporting information can be downloaded at https://www.mdpi.com/article/10.3390/toxics13090778/s1, Table S1: Multiple reaction monitoring (MRM) transitions for the analysis of chemicals in fishery products using LC-MSMS; Table S2. Method validation criteria for pesticide residues as suggested by the Codex Alimentarius Commission (CAC/GL 40-1993); Table S3 Matrix effects on 198 chemicals from flatfish and eel; Table S4. Recoveries and coefficients of variation (CVs) of 198 chemicals including 161 pesticides at three concentrations using the method suggested by MFDS; Table S5. Assessment of dietary exposure to chemicals in fishery products based on average consumption; Table S6. Acceptable daily intake (ADI) values for all analyzed compounds. Reference [52] is cited in the supplementary materials.
