# Supplementary material for: Nationwide Monitoring and Risk Assessment of Pesticide Residues in Fishery Products
Source: Toxics. 2025 Sep 14;13(9):778. doi: 10.3390/toxics13090778 (PMC12474335; doi:10.3390/toxics13090778)
Supplement: Supplementary file 1 [file toxics-13-00778-s001.zip › toxics-3852954-supplementary.pdf]

Table S1. Multiple reaction monitoring (MRM) transitions for the analysis of chemicals in fishery products using LC-MSMS

No.	Chemical	Ionization	Retention time (min)	Precursor ion (m/z)	Quantitative ion (m/z)	Collision energy (eV)	Qualitative ion (m/z)	Collision energy (eV)
1	Acephate	+	1.93	184.0	143.0	-10.0	95.2	-24.0
2	Acetamiprid	+	3.96	223.1	126.2	-21.0	99.1	-38.0
	N-Desmethyl-acetamiprid (IM-2-1)	+	3.97	209.0	126.0	-16.0	99.0	-37.0
3	Acynonapyr	+	10.35	505.1	342.0	-16.0	122.1	-30.0
4	Alachlor	+	6.81	270.2	162.2	-20.0	132.1	-39.0
5	Aldicarb	+	4.36	208.1	89.0	-16.0	116.1	-8.0
6	Amitraz	+	9.46	294.1	163.1	-20.0	122.1	-30.0
	2,4-Dimethylaniline	+	3.36	122.2	107.1	-20.0	77.0	-29.0
7	Atrazine	+	5.40	216.2	174.1	-17.0	96.1	-25.0
	Atrazine-desethyl (DEA)	+	4.18	188.2	146.1	-17.0	79.0	-26.0
	Atrazine-desisopropyl (DIA)	+	3.68	174.0	68.1	-28.0	79.1	-18.0
8	Azinphos-methyl	+	5.72	317.9	132.0	-13.0	159.9	-7.0
9	Azoxystrobin	+	5.90	404.1	372.2	-25.0	344.2	-35.0
10	Bendiocarb	+	4.72	224.1	109.2	-16.0	167.1	-9.0
11	Bensulfuron methyl	+	5.71	411.1	149.1	-21.0	182.0	-21.0
12	Benzovindiflupyr	+	7.37	398.0	342.1	-19.0	378.1	-15.0
13	Benzpyrimoxan	+	7.05	341.0	109.0	-50.0	87.1	-26.0
14	Bifenazate	+	6.56	301.2	170.2	-21.0	152.1	-42.0
	Bifenazate-diazene	+	8.08	299.1	197.1	-20.0	196.1	-15.0
15	Bioresmethrin	+	9.98	339.1	171.1	-16.0	128.1	-39.0
16	Boscalid	+	7.47	344.1	161.0	-25.0	69.0	-22.0
17	Brodifacoum	+	9.98	523.0	335.0	-22.0	178.1	-33.0
18	Buprofezine	+	8.60	306.1	57.1	-15.0	116.1	-20.0
19	Butamifos	+	7.73	333.2	152.1	-15.0	180.1	-15.0
20	Cafenstrole	+	6.49	351.0	100.2	-12.0	72.1	-27.0
21	Carbaryl	+	4.92	202.1	127.2	-25.0	145.2	-13.0
22	Carbendazim	+	3.36	192.0	132.1	-30.0	105.1	-34.0
23	Carbofuran	+	4.75	222.0	123.1	-21.0	165.2	-12.0
	3-Hydroxy carbofuran	+	3.96	238.0	181.1	-12.0	163.2	-20.0
24	Carbosulfan	+	10.18	381.3	76.1	-35.0	160.1	-16.0
25	Carfentrazone-ethyl	+	7.21	412.1	346.0	-24.0	383.9	-15.0
26	Carpropamide	+	7.45	334.0	139.0	-21.0	103.1	-42.0
27	Chlorantraniliprole	+	5.68	482.0	450.9	-17.0	112.0	-55.0
28	Chlorfenvinphos (*E*)	+	7.48	358.9	99.1	-33.0	155.2	-14.0
	Chlorfenvinphos (*Z*)	+	7.48	358.9	99.1	-33.0	155.2	-14.0
29	Chromafenozide	+	6.70	395.2	175.2	-18.0	147.1	-44.0
30	Clofentezine	+	7.81	303.0	138.0	-16.0	102.2	-36.0
31	Clomeprop	+	8.54	324.0	120.1	-21.0	203.0	-15.0

32	Clothianidin	+	3.80	250.0	169.1	-13.0	132.1	-16.0
33	Cumyluron	+	6.52	303.1	119.3	-21.0	73.1	-19.0
34	Cyantraniliprole	+	4.99	474.8	285.9	-16.0	443.9	-21.0
35	Cyclopyrimorate	+	6.39	390.1	70.0	-26.0	114.1	-15.0
36	Cyproconazole	+	6.49	292.1	70.2	-18.0	125.2	-30.0
37	Daimuron	+	6.40	269.1	151.2	-12.0	119.1	-21.0
38	Diazinon	+	7.44	305.2	100.0	-40.0	84.1	-34.0
39	Dichlorvos (DDVP)	+	4.66	239.0	182.2	-19.0	85.1	-16.0
40	Diclocymet (*E*)	+	7.03	313.1	137.0	-30.0	173.0	-17.0
	Diclocymet (*Z*)	+	7.03	313.1	137.0	-30.0	173.0	-17.0
41	Diflubenzuron	+	6.88	311.1	158.0	-16.0	141.0	-25.0
42	Dimethomorph (*E*)	+	6.06	388.1	301.1	-21.0	273.2	-15.0
	Dimethomorph (*Z*)	+	6.06	388.1	301.1	-21.0	273.2	-15.0
43	Dinotefuran	+	2.83	203.1	129.2	-13.0	113.2	-12.0
44	Disulfoton	+	4.07	275.0	61.1	-48.0	29.1	-52.0
	Disulfoton sulfone	+	5.21	307.0	125.0	-17.0	153.0	-12.0
	Disulfoton sulfoxide	+	5.15	291.0	185.1	-14.0	213.1	-10.0
	Demeton-S	+	5.82	259.0	89.1	-15.0	61.0	-32.0
	Demeton-S-sulfone	+	4.11	291.0	235.1	-15.0	263.2	-12.0
	Demeton-S-sulfoxide	+	4.06	275.1	81.1	-39.0	169.0	-17.0
45	Diuron	+	5.48	233.0	72.1	-22.0	46.1	-17.0
46	Edifenphos	+	7.37	311.0	109.0	-26.0	283.0	-19.0
47	Emamectin benzoate	+	8.65	886.3	158.2	-34.0	126.2	-40.0
48	Epoxyconazole	+	6.80	330.0	121.1	-21.0	123.0	-17.0
49	Esprocarb	+	8.55	266.2	71.1	-15.0	65.0	-54.0
50	Ethiofencarb	+	5.05	226.2	107.1	-15.0	164.1	-8.0
51	Ethiprole	+	6.01	397.1	350.9	-21.0	227.9	-52.0
	Ethiprole-sulfone	-	6.02	411.1	282.0	28.0	244.0	45.0
52	Ethoxyquin	+	5.73	218.1	174.1	-19.0	190.1	-18.0
	Ethoxyquin dimer	+	10.90	433.3	188.1	-42.0	375.1	-33.0
53	Etobenzanid	+	7.44	340.1	121.1	-34.0	149.1	-22.0
54	Etoxazole	+	9.26	360.2	304.2	-15.0	113.0	-35.0
55	Etrimfos	+	7.41	293.0	265.1	-17.0	125.1	-20.0
56	Famoxadone	+	7.51	392.1	331.2	-11.0	238.1	-17.0
57	Fenamidone	+	6.09	312.1	65.1	-49.0	92.1	-26.0
58	Fenarimol	+	6.74	331.1	268.0	-22.0	258.9	-25.0
59	Fenbuconazole	+	6.91	337.1	125.1	-29.0	194.1	-17.0
60	Fenhexamid	+	6.66	301.9	97.1	-24.0	55.1	-41.0
61	Fenobucarb	+	5.89	208.1	77.1	-38.0	95.1	-14.0
62	Fenoxasulfone	+	6.51	383.1	203.0	-14.0	366.1	-9.0
63	Fenpyroximate	+	9.47	422.1	366.1	-16.0	135.0	-32.0
64	Fensulfothion	+	5.41	309.0	281.1	-15.0	253.1	-18.0

	Fenthion (MPP)	+	3.97	279.1	264.0	-19.0	104.1	-28.0
	Fenthion oxon sulfone	+	4.05	295.1	78.1	-55.0	89.0	-52.0
65	Fenthion oxon sulfoxide	+	3.98	279.0	264.0	-20.0	104.1	-28.0
	Fenthion oxon	+	5.71	263.0	231.0	-15.0	120.9	-45.0
	Fenthion sulfone	+	4.96	311.1	125.1	-21.0	109.1	-31.0
	Fenthion sulfoxide	+	4.85	295.1	109.0	-30.0	278.0	-20.0
66	Fentrazamide	+	7.41	350.2	83.1	-24.0	197.2	-8.0
67	Ferimzone (*E*)	+	5.81	255.1	132.1	-21.0	124.1	-22.0
	Ferimzone (*Z*)	+	5.81	255.1	132.1	-21.0	124.1	-22.0
68	Flonicamid	+	3.31	230.0	203.0	-17.0	174.0	-18.0
	TFNA	+	4.32	192.1	138.1	-29.0	165.0	-21.0
69	Florpyrauxifen-benzyl	+	7.91	439.0	65.0	-40.0	421.2	-17.0
70	Fluazinam	-	8.74	463.0	415.5	21.0	397.5	17.0
71	Flubendiamide	-	7.19	680.8	253.9	29.0	274.2	18.0
72	Fludioxonil	+	6.19	265.9	228.9	-15.0	157.9	-32.0
73	Flufenoxuron	+	9.19	489.0	158.1	-22.0	141.1	-47.0
74	Flumioxazine	+	5.70	355.3	327.1	-20.0	298.9	-30.0
75	Fluopicolide	+	6.34	382.8	173.0	-22.0	109.1	-55.0
76	Flupyrimin	+	4.74	316.0	90.0	-33.0	99.0	-39.0
77	Fluralaner	+	8.27	556.1	400.0	-27.0	160.1	-46.0
78	Fluridone	+	5.71	330.0	259.1	-49.0	294.1	-46.0
79	Flutolanil	+	7.02	341.1	87.0	-27.0	159.0	-10.0
80	Fluxametamide	+	8.91	474.1	399.9	-25.0	160.0	-36.0
81	Fluxapyroxad	+	6.29	382.1	362.1	-14.0	342.1	-21.0
82	Furametpyr	+	5.30	334.1	290.1	-27.0	131.1	-33.0
83	Hexaconazole	+	7.58	314.0	70.2	-23.0	159.0	-31.0
84	Imazalil	+	5.02	296.9	159.1	-24.0	69.2	-19.0
82	Inpyrfluxam	+	6.89	349.2	331.0	-23.0	315.1	-34.0
86	Ipfencarbazone	+	7.37	427.1	198.0	-14.0	128.0	-40.0
87	Ipflufenoquin	+	7.10	348.2	330.1	-13.0	314.1	-35.0
88	Iprobenfos	+	7.21	289.1	205.0	-15.0	65.1	-45.0
89	Isoprothiolane	+	6.35	291.0	231.0	-16.0	145.0	-28.0
90	Isoxathion	+	7.69	314.0	105.0	-10.0	286.0	-10.0
91	Lufenuron	-	8.85	508.9	326.0	22.0	175.0	36.0
92	Malathion	+	7.87	330.8	127.2	-13.0	125.1	-28.0
93	Mefenacet	+	6.53	299.0	120.2	-20.0	148.2	-22.0
94	Metaflumizone (*E*)	+	8.74	507.1	178.0	-25.0	287.2	-25.0
	Metaflumizone (*Z*)	+	8.74	507.1	178.0	-24.0	287.2	-26.0
95	Metalaxyl	+	5.41	280.1	160.1	-23.0	220.1	-24.0
96	Methamidophos	+	0.93	142.0	94.1	-15.0	125.1	-20.0
97	Methidathion	+	5.62	303.0	85.1	-22.0	145.0	-20.0
98	Methiocarb	+	6.06	226.1	121.1	-17.0	169.2	-10.0

99	Methoxyfenozide	+	6.42	369.2	149.1	-18.0	133.2	-23.0
100	Metominostrobin	+	5.56	285.1	194.1	-15.0	166.1	-30.0
101	Metrafenone	+	7.82	408.9	209.2	-15.0	227.1	-21.0
102	Metyltetraprole	+	7.55	397.1	203.1	-25.0	91.1	-45.0
103	Molinate	+	6.49	188.0	126.2	-16.0	83.1	-19.0
104	Monocrotophos	+	3.51	224.0	127.0	-15.0	57.9	-26.0
105	Myclobutanil	+	7.21	289.1	205.0	-11.0	91.1	-10.0
106	Novaluron	+	8.31	492.8	158.1	-19.0	141.1	-45.0
107	Orysastrobin	+	6.34	392.2	116.1	-38.0	205.1	-25.0
108	Oxadiargyl	+	7.64	357.8	341.2	-11.0	223.0	-21.0
109	Oxathiapiprolin	+	6.35	540.2	500.1	-25.0	522.2	-27.0
110	Oxaziclomefone	+	8.41	376.1	133.1	-35.0	115.2	-43.0
111	Oxydemeton-methyl	+	3.28	246.9	169.0	-16.0	109.0	-30.0
112	Penconazole	+	7.21	284.2	159.0	-27.0	123.0	-50.0
113	Pencycuron	+	7.88	328.9	125.2	-10.0	89.0	-35.0
114	Pendimethalin	+	9.01	282.1	212.1	-12.0	134.1	-30.0
115	Penoxsulam	+	4.96	484.0	195.1	-27.0	164.1	-32.0
116	Phorate	+	3.96	260.9	75.1	-16.0	125.0	-16.0
	Phorate oxon sulfone	+	4.09	277.0	111.0	-24.0	127.0	-16.0
	Phorate oxon sulfoxide	+	3.98	261.0	111.1	-22.0	243.0	-9.0
	Phorate oxon	+	5.62	245.0	75.1	-11.0	47.0	-33.0
	Phorate sulfone	+	5.18	293.0	171.0	-11.0	143.0	-22.0
	Phorate sulfoxide	+	5.15	277.1	171.0	-14.0	142.9	-10.0
117	Phoxim	+	7.66	299.0	77.1	-29.0	129.2	-11.0
118	Pirimicarb	+	4.66	239.1	72.1	-28.0	182.2	-23.0
119	Probenazole	+	4.55	224.1	41.1	-14.0	196.0	-10.0
120	Profenofos	+	8.44	372.8	302.9	-19.0	128.0	-47.0
121	Propamocarb	+	4.31	189.8	163.0	-22.0	136.1	-10.0
122	Propiconazole	+	7.47	342.1	69.0	-22.0	41.1	-33.0
123	Propoxur	+	4.67	210.1	65.1	-34.0	168.1	-8.0
124	Propyrisulfuron	+	6.34	455.8	261.0	-18.0	218.1	-20.0
125	Prosulfocarb	+	8.29	252.1	91.2	-24.0	65.1	-55.0
126	Pydiflumetofen	+	7.82	426.1	406.1	-13.0	218.1	-20.0
127	Pyraclostrobin	+	7.65	388.1	149.0	-27.0	296.2	-15.0
128	Pyribencarb	+	5.80	362.1	207.0	-20.0	146.1	-24.0
129	Pyributicarb	+	8.89	331.2	190.1	-17.0	105.1	-36.0
130	Pyridaben	+	9.77	365.1	147.3	-15.0	309.2	-20.0
131	Pyriofenone	+	7.84	366.3	209.1	-25.0	166.1	-37.0
132	Pyriproxyfen	+	8.80	322.0	185.1	-22.0	227.1	-15.0
133	Pyroquilon	+	4.66	174.0	77.0	-43.0	117.1	-15.0
134	Quinoclamine	+	5.92	208.1	77.0	-36.0	95.1	-15.0
135	Saflufenacil	+	5.77	501.0	198.0	-44.0	349.0	-28.0

136	Sedaxane	+	6.51	332.2	292.1	-16.0	312.2	-14.0
137	Sethoxydim	+	8.61	328.1	178.2	-20.0	282.2	-11.0
138	Simazine	+	4.81	202.0	132.1	-17.0	96.2	-22.0
	Shimazine-2-hydroxy (OH-Simazine)	+	3.11	184.1	114.1	-18.0	69.1	-33.0
139	Spinetoram J	+	8.07	748.5	98.1	-54.0	115.3	-49.0
	Spinetoram L	+	8.48	760.3	142.2	-30.0	98.2	-55.0
140	Spinosyn A	+	7.59	732.2	142.2	-29.0	98.0	-55.0
	Spinosyn D	+	8.01	746.2	142.2	-29.0	98.0	-55.0
141	Spiromesifen	+	9.18	371.3	273.2	-16.0	255.2	-25.0
142	Spirotetramat	+	6.69	374.0	216.0	-34.0	302.1	-12.0
143	Sulfosulfuron	+	5.78	471.1	211.0	-14.0	218.0	-29.0
144	Sulfoxaflor	+	4.06	278.0	174.2	-10.0	154.1	-26.0
145	Tebuconazole	+	7.29	308.0	70.0	-22.0	125.0	-37.0
146	Tebufenozide	+	7.15	353.2	297.2	-8.0	105.1	-42.0
147	Tebufloquin	+	7.34	290.2	233.1	-34.0	218.1	-41.0
	Tebufloquin M1	+	6.00	248.2	218.2	-35.0	191.0	-37.0
148	Teflubenzuron	-	8.67	378.8	339.0	13.0	358.9	8.0
149	Terbuthylazine	+	6.23	230.2	174.1	-26.0	132.0	-24.0
	Terbuthylazine-2-hydroxy (OH-TER)	+	3.90	212.2	156.0	-16.0	86.2	-24.0
	Terbuthylazine-desethyl (DE-TER)	+	4.93	202.0	146.0	-16.0	79.1	-26.0
	Terbuthylazine-desethyl-2-hydroxy (OH-DE-TER)	+	2.86	184.1	128.1	-16.0	85.9	-24.0
150	Tetraniliprole	+	5.50	545.0	356.1	-14.0	375.9	-26.0
151	Thiabendazole	+	3.80	202.1	65.1	-46.0	77.1	-52.0
	5-Hydroxy thiabendazole	+	3.35	218.1	191.1	-25.0	81.0	-45.0
152	Thiacloprid	+	4.17	253.0	99.0	-43.0	90.1	-39.0
153	Thiamethoxam	+	3.39	292.0	211.1	-12.0	181.1	-22.0
154	Thiobencarb	+	7.79	258.0	125.2	-22.0	89.1	-49.0
155	Tiadinil	-	6.48	265.8	71.1	23.0	238.0	12.0
156	Tolprocarb	+	6.03	347.1	91.0	-30.0	65.0	-40.0
157	Trichlorfon (Metrifonate)	+	3.93	256.8	109.1	-20.0	221.0	-11.0
158	Tricyclazole	+	4.32	190.0	136.1	-26.0	163.1	-15.0
159	Trifloxystrobin	+	8.18	409.2	116.0	-23.0	131.1	-30.0
160	Triflumezopyrim	+	7.59	399.1	203.1	-15.0	146.1	-20.0
161	Warfarin	+	6.20	309.1	121.0	-41.0	147.1	-16.0

Table S2. Method validation criteria for pesticide residues as suggested by the Codex Alimentarius Commission (CAC/GL 40-1993)

Concentration	Coefficient of variation (%)	Recovery (%)
≤0.001 mg/kg	35	50–120
>0.001 mg/kg to ≤0.01 mg/kg	30	60–120
>0.01 mg/kg to ≤0.1 mg/kg	20	70–120
>0.1 mg/kg to ≤1 mg/kg	15	70–110
> 1 mg/kg	10	70–110

Table S3 Matrix effects on 198 chemicals from flatfish and eel

		Chemical	Matrix effect (%)	
			Flatfish	Eel
1	1	Acephate	5.7	6.3
2	2	Acetamiprid	4.6	2.5
	3	*N*-Desmethyl-acetamiprid (IM-2-1)	0.9	16.5
3	4	Acynonapyr	1102.8	77423.0
4	5	Alachlor	2.9	15.2
5	6	Aldicarb	6.6	5.7
6	7	Amitraz	24.4	20.7
	8	2,4-Dimethylaniline	2.7	8.0
7	9	Atrazine	7.1	7.1
	10	Atrazine-desethyl (DEA)	0.4	0.3
	11	Atrazine-desisopropyl (DIA)	3.7	5.3
8	12	Azinphos-methyl	1.9	5.0
9	13	Azoxystrobin	21.7	3.5
10	14	Bendiocarb	5.6	7.2
11	15	Bensulfuron methyl	12.5	6.9
12	16	Benzovindiflupyr	8.5	27.2
13	17	Benzpyrimoxan	6.5	10.8
14	18	Bifenazate	1.9	13.2
	19	Bifenazate-diazene	1.1	8.8
15	20	Bioresmethrin	59.3	16.3
16	21	Boscalid	4.1	7.4
17	22	Brodifacoum	42.9	8.6
18	23	Buprofezine	9.0	7.0
19	24	Butamifos	6.3	0.2
20	25	Cafenstrole	2.9	19.2
21	26	Carbaryl	0.4	7.7
22	27	Carbendazim	0.5	2.1
23	28	Carbofuran	10.9	2.4
	29	3-Hydroxy carbofuran	8.4	6.4
24	30	Carbosulfan	10.8	6.7
25	31	Carfentrazone-ethyl	0.5	19.2
26	32	Carpropamide	23.0	2.2

27	33	Chlorantraniliprole	3.0	3.3
28	34	Chlorfenvinphos (*E*)	3.1	5.0
	35	Chlorfenvinphos (*Z*)	12.5	12.3
29	36	Chromafenozide	4.6	1.7
30	37	Clofentezine	1.4	1.8
31	38	Clomeprop	2.2	3.5
32	39	Clothianidin	5.4	8.0
33	40	Cumyluron	11.4	8.3
34	41	Cyantraniliprole	9.1	1.6
35	42	Cyclopyrimorate	15.4	20.4
36	43	Cyproconazole	8.9	3.3
37	44	Daimuron	1.4	20.4
38	45	Diazinon	13.1	2.1
39	46	Dichlorvos (DDVP)	1.8	3.9
40	47	Diclocymet (*E*)	1.9	1.6
	48	Diclocymet (*Z*)	33.6	31.4
41	49	Diflubenzuron	5.7	8.9
42	50	Dimethomorph (*E*)	5.7	5.4
	51	Dimethomorph (*Z*)	9.6	4.0
43	52	Dinotefuran	4.2	1.6
44	53	Disulfoton	3.7	5.3
	54	Disulfoton sulfone	0.8	3.7
	55	Disulfoton sulfoxide	2.9	6.0
	56	Demeton-S	5.0	7.8
	57	Demeton-S-sulfone	6.9	6.4
	58	Demeton-S-sulfoxide	6.8	14.4
45	59	Diuron	0.6	2.3
46	60	Edifenphos	3.3	7.7
47	61	Emamectin benzoate	18.2	2.7
48	62	Epoxyconazole	27.8	36.1
49	63	Esprocarb	6.7	3.7
50	64	Ethiofencarb	14.6	4.3
51	65	Ethiprole	5.1	16.9
	66	Ethiprole-sulfone	1.8	2.7
52	67	Ethoxyquin	8.7	4.3
	68	Ethoxyquin dimer	17.6	11.5

53	69	Etobenzanid	32.1	6.8
54	70	Etoxazole	5.9	5.3
55	71	Etrimfos	11.1	4.5
56	72	Famoxadone	8.5	5.3
57	73	Fenamidone	15.4	1.5
58	74	Fenarimol	4.2	8.2
59	75	Fenbuconazole	6.6	4.5
60	76	Fenhexamid	3.1	7.5
61	77	Fenobucarb	6.4	3.3
62	78	Fenoxasulfone	2.7	3.9
63	79	Fenpyroximate	18.4	6.4
64	80	Fensulfothion	6.8	3.9
65	81	Fenthion (MPP)	2.1	1.3
	82	Fenthion oxon sulfone	0.7	1.1
	83	Fenthion oxon sulfoxide	4.7	11.2
	84	Fenthion oxon	12.8	1.0
	85	Fenthion sulfone	0.2	4.4
	86	Fenthion sulfoxide	4.1	0.5
66	87	Fentrazamide	1.0	5.4
67	88	Ferimzone (*E*)	11.8	0.1
	89	Ferimzone (*Z*)	2.6	8.4
68	90	Flonicamid	2.4	3.2
	91	TFNA	2.5	8.2
69	92	Florpyrauxifen-benzyl	7.2	2.4
70	93	Fluazinam	17.7	3.8
71	94	Flubendiamide	12.3	13.9
72	95	Fludioxonil	5.5	8.0
73	96	Flufenoxuron	6.4	15.8
74	97	Flumioxazine	21.5	10.1
75	98	Fluopicolide	6.9	7.5
76	99	Flupyrimin	1.3	15.5
77	100	Fluralaner	1.4	2.8
78	101	Fluridone	8.0	16.8
79	102	Flutolanil	1.7	6.6
80	103	Fluxametamide	21.3	21.8
81	104	Fluxapyroxad	14.7	6.4

82	105	Furametpyr	2.4	10.4
83	106	Hexaconazole	2.3	6.5
84	107	Imazalil	4.0	0.4
85	108	Inpyrfluxam	5.2	1.9
86	109	Ipfencarbazone	5.5	2.4
87	110	Ipflufenoquin	10.9	1.3
88	111	Iprobenfos	6.9	8.7
89	112	Isoprothiolane	11.1	10.5
90	113	Isoxathion	10.1	6.5
91	114	Lufenuron	30.6	18.1
92	115	Malathion	0.5	8.4
93	116	Mefenacet	4.3	4.5
94	117	Metaflumizone (*E*)	2.9	2.1
	118	Metaflumizone (*Z*)	1.3	4.0
95	119	Metalaxyl	13.6	13.1
96	120	Methamidophos	57.9	15.9
97	121	Methidathion	6.1	9.3
98	122	Methiocarb	2.5	2.0
99	123	Methoxyfenozide	18.7	3.6
100	124	Metominostrobin	44.2	4.2
101	125	Metrafenone	20.0	11.0
102	126	Metyltetraprole	8.2	8.1
103	127	Molinate	1.3	0.1
104	128	Monocrotophos	0.5	4.1
105	129	Myclobutanil	0.4	0.2
106	130	Novaluron	0.2	9.0
107	131	Orysastrobin	1.5	0.3
108	132	Oxadiargyl	9.0	10.5
109	133	Oxathiapiprolin	5.4	6.0
110	134	Oxaziclomefone	2.8	5.0
111	135	Oxydemeton-methyl	8.4	18.8
112	136	Penconazole	5.5	4.7
113	137	Pencycuron	12.2	1.5
114	138	Pendimethalin	1.2	5.2
115	139	Penoxsulam	7.3	8.4
116	140	Phorate	6.1	7.6

	141	Phorate oxon sulfone	0.8	13.3
	142	Phorate oxon sulfoxide	1.7	2.4
	143	Phorate oxon	1.5	0.1
	144	Phorate sulfone	3.5	12.7
	145	Phorate sulfoxide	1.5	2.6
117	146	Phoxim	5.9	2.6
118	147	Pirimicarb	1.3	3.6
119	148	Probenazole	8.1	11.3
120	149	Profenofos	18.7	4.8
121	150	Propamocarb	2.6	2.9
122	151	Propiconazole	3.7	0.4
123	152	Propoxur	2.6	1.6
124	153	Propyrisulfuron	4.0	0.4
125	154	Prosulfocarb	9.8	2.8
126	155	Pydiflumetofen	2.6	1.5
127	156	Pyraclostrobin	9.0	13.9
128	157	Pyribencarb	3.2	2.2
129	158	Pyributicarb	9.9	5.7
130	159	Pyridaben	5.3	7.4
131	160	Pyriofenone	2.1	11.6
132	161	Pyriproxyfen	9.4	3.1
133	162	Pyroquilon	0.2	7.8
134	163	Quinoclamine	20.1	2.8
135	164	Saflufenacil	2.1	1.4
136	165	Sedaxane	12.1	23.2
137	166	Sethoxydim	5.4	6.1
138	167	Simazine	6.3	11.0
	168	Shimazine-2-hydroxy (OH-Simazine)	10.3	18.5
139	169	Spinetoram J	1.0	10.9
	170	Spinetoram L	2.0	3.8
140	171	Spinosyn A	0.7	4.8
	172	Spinosyn D	14.1	11.9
141	173	Spiromesifen	13.1	2.5
142	174	Spirotetramat	8.2	15.4
143	175	Sulfosulfuron	6.8	1.7

144	176	Sulfoxaflor	10.1	4.5
145	177	Tebuconazole	2.7	2.8
146	178	Tebufenozide	9.9	0.4
147	179	Tebufloquin	5.2	7.8
	180	Tebufloquin M1	21.9	10.2
148	181	Teflubenzuron	19.2	11.8
149	182	Terbuthylazine	19.1	16.1
	183	Terbuthylazine-2-hydroxy (OH-TER)	5.0	2.0
	184	Terbuthylazine-desethyl (DE-TER)	3.9	0.4
	185	Terbuthylazine-desethyl-2-hydroxy (OH-DE-TER)	10.0	3.2
150	186	Tetraniliprole	0.1	7.8
151	187	Thiabendazole	0.3	7.5
	188	5-Hydroxy thiabendazole	4.1	1.1
152	189	Thiacloprid	2.3	3.0
153	190	Thiamethoxam	2.6	1.7
154	191	Thiobencarb	0.7	1.4
155	192	Tiadinil	18.5	8.9
156	193	Tolprocarb	2.5	15.2
157	194	Trichlorfon (Metrifonate)	20.1	12.2
158	195	Tricyclazole	5.1	0.8
159	196	Trifloxystrobin	0.5	5.0
160	197	Triflumezopyrim	0.4	8.5
161	198	Warfarin	15.9	5.2

Table S4. Recoveries and coefficients of variation (CVs) of 198 chemicals including 161 pesticides at three concentrations using the method suggested by MFDS

		Chemical	Concentration (mg/kg)	Flatfish		Eel	
				Recovery (%)	CV (%)	Recovery (%)	CV (%)
1	1	Acephate	0.01	96.5	8.6	106.2	1.7
			0.02	102.9	2.5	103.2	1.1
			0.1	103.3	1.7	100.6	2.8
2	2	Acetamiprid	0.01	68.5	3.6	69.3	2.9
			0.02	101.5	2.9	100.2	1.1
			0.1	109.1	2.2	110.9	1.4
	3	*N*-Desmethyl-acetamiprid (IM-2-1)	0.01	68.0	5.5	62.6	4.0
			0.02	100.3	2.4	101.8	2.4
			0.1	107.9	1.3	111.1	2.0
3	4	Acynonapyr	0.01	113.9	1.7	-	-
			0.02	102.8	0.7	90.4	1.4
			0.1	101.3	0.9	79.3	2.9
4	5	Alachlor	0.01	81.1	7.7	86.9	5.5
			0.02	102.7	4.1	104.8	2.0
			0.1	109.7	3.9	107.0	2.0
5	6	Aldicarb	0.01	98.9	4.9	85.6	7.6
			0.02	102.9	2.2	98.4	5.1
			0.1	102.1	3.1	100.7	3.6
6	7	Amitraz	0.01	92.5	2.5	96.2	3.9
			0.02	99.8	1.9	101.5	1.6
			0.1	100.6	1.9	100.5	2.1
	8	2,4-Dimethylaniline	0.01	100.1	9.4	96.0	20.1
			0.02	103.7	13.6	95.0	17.5
			0.1	103.2	6.0	100.3	2.8
7	9	Atrazine	0.01	80.2	4.5	86.1	10.3
			0.02	97.9	6.8	101.9	2.6
			0.1	107.4	5.9	104.7	8.3
	10	Atrazine-desethyl (DEA)	0.01	85.2	3.3	74.9	8.6
			0.02	100.6	5.1	100.9	3.6
			0.1	105.6	1.7	107.9	3.1
	11	Atrazine-desisopropyl (DIA)	0.01	93.5	6.8	80.4	3.7
			0.02	101.6	2.7	98.1	2.5
			0.1	104.0	2.1	104.3	1.7
8	12	Azinphos-methyl	0.01	71.1	12.4	63.8	3.5
			0.02	108.0	5.5	97.2	8.8
			0.1	117.1	0.8	115.4	2.8
9	13	Azoxystrobin	0.01	95.4	5.1	92.6	3.8
			0.02	99.4	2.8	101.7	5.2
			0.1	100.0	4.0	100.2	4.6
10	14	Bendiocarb	0.01	78.6	2.8	81.4	4.7
			0.02	99.7	6.2	100.1	3.6
			0.1	110.3	2.4	111.8	2.3
11	15	Bensulfuron methyl	0.01	77.7	10.4	79.0	10.2
			0.02	96.6	5.9	101.3	5.7
			0.1	105.3	6.6	99.7	7.2
12	16	Benzovindiflupyr	0.01	65.4	4.4	64.3	3.4
			0.02	98.2	6.0	102.4	13.3
			0.1	113.5	3.5	116.5	2.5
13	17	Benzpyrimoxan	0.01	96.6	4.0	87.5	4.7
			0.02	105.5	4.7	103.4	4.2
			0.1	104.0	7.8	103.0	4.2
14	18	Bifenazate	0.01	93.6	4.5	87.1	7.9
			0.02	92.7	7.2	104.5	8.1
			0.1	96.6	2.5	105.8	6.3
	19	Bifenazate-diazene	0.01	88.9	6.5	100.4	5.4
			0.02	98.0	8.4	102.8	4.7
			0.1	107.9	6.1	107.1	5.3
15	20	Bioresmethrin	0.01	102.7	4.6	98.0	2.5
			0.02	101.8	0.8	99.3	3.0
			0.1	100.2	1.9	99.9	1.4
16	21	Boscalid	0.01	96.7	7.9	94.5	4.2
			0.02	101.1	6.5	103.7	4.5

			0.1	100.5	7.2	108.4	5.7
17	22	Brodifacoum	0.01	104.7	3.5	100.8	2.3
			0.02	100.5	1.8	99.9	2.4
			0.1	99.9	1.3	100.6	1.9
18	23	Buprofezine	0.01	87.5	1.9	82.6	1.9
			0.02	101.2	2.9	99.7	3.8
			0.1	102.3	1.0	107.0	3.1
19	24	Butamifos	0.01	91.2	8.2	83.5	5.7
			0.02	100.3	7.2	105.2	5.9
			0.1	105.2	5.2	106.6	3.3
20	25	Cafenstrole	0.01	72.1	17.5	64.9	4.6
			0.02	93.5	5.3	98.0	11.0
			0.1	107.4	5.5	110.7	5.1
21	26	Carbaryl	0.01	91.1	8.5	86.1	7.2
			0.02	102.5	4.6	98.1	3.8
			0.1	107.8	3.5	102.5	2.8
22	27	Carbendazim	0.01	92.3	3.3	95.5	4.4
			0.02	101.9	1.7	99.4	4.1
			0.1	103.7	0.7	102.9	2.0
23	28	Carbofuran	0.01	98.2	8.0	94.6	9.7
			0.02	101.0	7.7	106.7	3.7
			0.1	101.5	5.7	96.6	3.8
	29	3-Hydroxy carbofuran	0.01	79.2	6.1	79.5	3.0
			0.02	102.3	2.1	97.7	2.7
			0.1	107.7	3.2	107.3	2.1
24	30	Carbosulfan	0.01	98.2	1.7	98.8	2.4
			0.02	101.0	2.3	102.1	0.6
			0.1	101.5	0.5	101.6	2.2
25	31	Carfentrazone-ethyl	0.01	78.2	24.4	110.6	8.0
			0.02	94.6	14.7	111.6	6.2
			0.1	99.8	10.4	111.9	7.6
26	32	Carpropamide	0.01	84.1	7.9	80.6	5.0
			0.02	102.6	4.1	99.6	3.8
			0.1	108.1	2.7	104.5	2.3
27	33	Chlorantraniliprole	0.01	91.0	9.4	71.4	12.6
			0.02	97.1	12.8	91.1	8.0
			0.1	106.5	5.7	108.3	7.0
28	34	Chlorfenvinphos (*E*)	0.01	90.1	5.3	93.0	3.9
			0.02	102.2	2.0	103.6	4.2
			0.1	101.9	3.5	102.3	4.1
	35	Chlorfenvinphos (*Z*)	0.01	101.2	9.6	96.5	7.9
			0.02	100.5	5.0	100.1	9.1
			0.1	98.7	3.1	101.6	1.9
29	36	Chromafenozide	0.01	75.2	5.8	88.5	7.1
			0.02	101.5	5.7	98.4	7.1
			0.1	107.6	5.1	102.2	3.2
30	37	Clofentezine	0.01	77.5	14.0	78.9	6.2
			0.02	90.9	6.9	94.9	5.9
			0.1	105.5	7.0	103.0	4.7
31	38	Clomeprop	0.01	92.4	5.0	90.6	5.5
			0.02	102.4	3.3	96.4	4.3
			0.1	103.5	3.6	101.7	4.0
32	39	Clothianidin	0.01	83.6	5.0	78.6	3.6
			0.02	103.0	3.7	104.4	1.7
			0.1	103.3	6.5	108.1	2.7
33	40	Cumyluron	0.01	79.7	17.7	67.2	8.2
			0.02	109.1	3.5	105.6	11.7
			0.1	103.1	3.6	112.7	5.9
34	41	Cyantraniliprole	0.01	107.7	4.9	92.7	4.3
			0.02	108.4	4.0	107.1	6.4
			0.1	102.7	4.2	105.9	2.9
35	42	Cyclopyrimorate	0.01	89.5	6.1	76.9	9.4
			0.02	103.9	5.1	93.5	8.8
			0.1	113.3	4.3	107.8	6.6
36	43	Cyproconazole	0.01	91.8	6.4	82.9	9.9
			0.02	97.4	6.5	103.8	5.5

			0.1	102.6	3.1	102.2	5.7
37	44	Dymron	0.01	63.2	3.5	64.9	8.3
			0.02	83.1	11.9	94.8	10.3
			0.1	101.8	9.4	107.7	7.2
38	45	Diazinon	0.01	93.6	7.5	71.6	7.9
			0.02	100.4	6.9	95.3	3.7
			0.1	103.2	2.2	109.0	3.5
39	46	Dichlorvos (DDVP)	0.01	99.1	3.2	94.5	4.7
			0.02	99.9	2.4	97.3	3.2
			0.1	99.8	1.0	98.6	2.3
40	47	Diclocymet (*E*)	0.01	80.3	23.4	76.0	19.8
			0.02	86.5	11.2	88.1	11.6
			0.1	103.3	10.5	105.4	9.2
	48	Diclocymet (*Z*)	0.01	82.2	21.4	83.6	18.0
			0.02	95.8	18.4	96.7	12.0
			0.1	104.2	2.6	102.5	7.4
41	49	Diflubenzuron	0.01	93.9	9.2	95.1	3.0
			0.02	100.1	5.2	104.6	5.5
			0.1	96.7	8.1	107.5	2.9
42	50	Dimethomorph (*E*)	0.01	96.5	7.9	102.8	7.1
			0.02	109.6	6.3	110.0	4.2
			0.1	100.9	9.5	109.0	4.3
	51	Dimethomorph (*Z*)	0.01	92.2	10.6	71.2	12.8
			0.02	105.1	6.3	99.1	12.7
			0.1	102.7	6.5	104.3	5.3
43	52	Dinotefuran	0.01	82.9	4.2	88.5	6.4
			0.02	96.3	6.4	96.3	5.2
			0.1	102.9	5.1	99.1	4.5
44	53	Disulfoton	0.01	86.8	5.3	89.8	6.2
			0.02	100.2	5.4	100.1	1.7
			0.1	105.6	2.7	104.1	2.5
	54	Disulfoton sulfone	0.01	89.3	7.5	77.3	7.6
			0.02	103.6	5.6	102.9	3.1
			0.1	103.0	1.4	109.6	1.8
	55	Disulfoton sulfoxide	0.01	76.6	4.3	95.5	4.0
			0.02	99.3	3.7	102.7	3.0
			0.1	105.6	1.5	107.9	1.5
	56	Demeton-S	0.01	65.0	2.0	64.7	5.7
			0.02	97.2	3.2	94.5	2.2
			0.1	113.8	2.1	103.5	5.1
	57	Demeton-S-sulfone	0.01	82.4	5.1	78.7	3.5
			0.02	99.4	3.4	97.5	1.7
			0.1	103.8	0.7	107.4	2.0
	58	Demeton-S-sulfoxide	0.01	91.0	3.5	93.8	2.4
			0.02	100.8	1.7	102.7	3.3
			0.1	105.2	2.3	105.5	0.8
45	59	Diuron	0.01	81.0	8.1	98.4	3.9
			0.02	100.3	6.2	106.4	2.9
			0.1	105.6	3.3	109.0	2.9
46	60	Edifenphos	0.01	86.5	3.7	73.0	9.9
			0.02	98.1	4.2	96.1	4.8
			0.1	107.4	6.0	108.7	2.6
47	61	Emamectin benzoate	0.01	94.4	1.4	95.1	2.6
			0.02	101.4	1.2	98.6	3.2
			0.1	101.9	1.5	103.9	2.9
48	62	Epoxyconazole	0.01	97.7	8.0	90.6	7.6
			0.02	100.6	4.4	100.5	5.1
			0.1	99.0	7.9	99.8	5.1
49	63	Esprocarb	0.01	87.5	12.6	83.5	11.6
			0.02	94.3	6.1	107.6	6.0
			0.1	102.2	4.1	108.7	3.2
50	64	Ethiofencarb	0.01	97.6	6.1	80.8	8.7
			0.02	102.7	5.6	88.6	3.9
			0.1	99.7	4.8	98.9	6.8
51	65	Ethiprole	0.01	94.3	14.2	66.9	10.0
			0.02	96.9	7.3	98.8	8.9

			0.1	101.7	5.9	113.3	4.0
	66	Ethiprole-sulfone	0.01	114.4	7.4	102.8	20.2
			0.02	108.7	9.7	112.9	5.0
			0.1	98.4	9.7	97.5	3.7
52	67	Ethoxyquin	0.01	76.3	13.2	83.5	12.6
			0.02	97.1	5.8	107.4	6.2
			0.1	102.9	5.4	104.2	7.3
	68	Ethoxyquin dimer	0.01	105.0	13.2	99.1	9.6
			0.02	108.7	4.6	95.4	7.8
			0.1	100.4	8.1	95.6	11.4
53	69	Etobenzanid	0.01	84.1	12.7	88.9	15.7
			0.02	99.0	8.1	108.4	4.0
			0.1	104.7	3.3	102.4	5.7
54	70	Etoxazole	0.01	104.9	1.4	96.5	6.1
			0.02	104.5	3.2	98.3	4.8
			0.1	98.2	2.3	102.4	5.7
55	71	Etrimfos	0.01	69.7	7.5	88.1	5.9
			0.02	99.6	2.8	103.1	5.9
			0.1	107.7	3.5	107.2	2.7
56	72	Famoxadone	0.01	89.3	6.1	84.5	9.7
			0.02	98.1	3.4	96.4	5.0
			0.1	103.9	5.1	108.4	2.7
57	73	Fenamidone	0.01	97.1	6.0	72.4	7.2
			0.02	106.3	4.4	101.7	10.2
			0.1	104.8	2.5	107.6	4.4
58	74	Fenarimol	0.01	75.4	16.7	86.2	18.6
			0.02	98.4	9.5	98.5	18.3
			0.1	108.6	4.1	109.6	1.5
59	75	Fenbuconazole	0.01	94.2	8.2	96.5	12.2
			0.02	97.4	11.9	101.7	5.3
			0.1	100.6	5.9	99.1	4.4
60	76	Fenhexamid	0.01	97.1	4.8	90.6	7.0
			0.02	99.0	10.0	100.0	6.7
			0.1	102.8	7.5	99.9	4.5
61	77	Fenobucarb	0.01	96.5	9.9	71.5	11.5
			0.02	102.3	2.9	84.4	8.3
			0.1	101.4	5.6	96.9	5.8
62	78	Fenoxasulfone	0.01	74.0	13.5	69.2	11.2
			0.02	97.5	12.8	109.5	6.5
			0.1	106.6	3.9	113.6	7.1
63	79	Fenpyroximate	0.01	99.8	1.3	106.2	1.1
			0.02	100.5	1.8	101.2	4.1
			0.1	99.1	2.2	99.7	2.9
64	80	Fensulfothion	0.01	80.7	2.9	67.9	6.2
			0.02	101.2	3.2	99.7	6.5
			0.1	103.4	3.3	107.9	3.5
65	81	Fenthion (MPP)	0.01	78.9	1.6	76.3	5.6
			0.02	100.0	4.5	100.2	2.7
			0.1	105.0	1.5	109.1	2.4
	82	Fenthion oxon sulfone	0.01	66.3	7.5	74.3	17.7
			0.02	98.9	6.2	103.5	3.0
			0.1	111.0	2.6	113.2	2.4
	83	Fenthion oxon sulfoxide	0.01	75.1	3.6	68.7	7.6
			0.02	100.6	4.9	101.0	6.1
			0.1	108.0	2.4	112.1	2.6
	84	Fenthion oxon	0.01	88.7	3.9	88.9	12.6
			0.02	97.3	3.8	100.4	9.0
			0.1	83.9	2.3	84.2	3.3
	85	Fenthion sulfone	0.01	69.4	11	90.8	6.8
			0.02	92.2	16	83.2	10
			0.1	101.1	7.1	104.2	6.9
	86	Fenthion sulfoxide	0.01	103.3	14.9	78.8	10.9
			0.02	102.5	7.8	103.0	6.5
			0.1	103.8	3.4	109.4	2.7
66	87	Fentrazamide	0.01	93.8	5.9	92.8	5.7
			0.02	99.2	3.1	102.4	6.1

			0.1	98.9	4.0	107.8	4.2
67	88	Ferimzone (*E*)	0.01	74.3	14.5	74.0	11.0
			0.02	102.7	7.5	100.8	12.5
			0.1	106.4	4.9	105.8	10.9
	89	Ferimzone (*Z*)	0.01	95.1	2.4	85.1	1.8
			0.02	98.9	2.8	95.3	4.5
			0.1	99.7	3.5	98.8	3.8
68	90	Flonicamid	0.01	93.6	1.7	103.5	3.5
			0.02	97.5	3.8	100.8	2.5
			0.1	100.5	0.8	97.4	2.3
	91	TFNA	0.01	98.2	14.3	105.7	6.1
			0.02	112.3	6.3	105.0	9.3
			0.1	105.5	5.5	95.2	11.6
69	92	Florpyrauxifen-benzyl	0.01	82.6	9.6	82.3	9.7
			0.02	101.9	6.1	100.4	2.8
			0.1	103.8	2.5	104.4	3.1
70	93	Fluazinam	0.01	95.4	14.9	84.5	19.4
			0.02	101.6	12.9	108.4	6.0
			0.1	102.9	4.8	101.6	7.0
71	94	Flubendiamide	0.01	89.0	5.6	101.3	4.3
			0.02	97.9	5.5	103.8	3.4
			0.1	105.2	1.2	99.2	3.9
72	95	Fludioxonil	0.01	114.8	4.9	107.6	15.0
			0.02	106.9	8.6	111.5	6.9
			0.1	94.2	6.0	96.5	4.7
73	96	Flufenoxuron	0.01	90.5	10.5	74.7	16.3
			0.02	112.0	6.8	107.0	4.9
			0.1	108.1	3.3	114.9	1.9
74	97	Flumioxazine	0.01	97.7	3.0	91.6	3.7
			0.02	103.7	3.9	95.8	2.4
			0.1	100.2	2.6	101.6	3.8
75	98	Fluopicolide	0.01	105.6	10.5	102.8	19.8
			0.02	96.4	12.5	95.5	18.5
			0.1	88.6	6.0	100.6	6.9
76	99	Flupyrimin	0.01	62.5	3.9	63.0	3.3
			0.02	102.2	7.0	101.2	5.3
			0.1	116.8	1.9	115.3	3.4
77	100	Fluralaner	0.01	81.0	14.3	84.9	9.6
			0.02	104.8	10.4	103.9	5.7
			0.1	111.2	2.4	107.9	5.0
78	101	Fluridone	0.01	106.5	12.2	109.9	7.8
			0.02	100.4	10.4	101.6	3.2
			0.1	94.1	3.3	98.1	5.8
79	102	Flutolanil	0.01	73.0	11.8	80.9	8.6
			0.02	98.3	11.9	100.7	14.6
			0.1	103.2	7.0	112.9	6.8
80	103	Fluxametamide	0.01	97.5	4.3	92.8	8.3
			0.02	105.6	11.5	104.8	8.4
			0.1	107.9	4.8	104.4	8.0
81	104	Fluxapyroxad	0.01	78.0	16.1	114.1	3.5
			0.02	89.0	16.9	90.1	6.3
			0.1	95.4	7.1	91.5	8.0
82	105	Furametpyr	0.01	71.6	11.8	64.0	4.5
			0.02	100.7	11.4	99.4	4.9
			0.1	111.1	6.1	109.2	7.5
83	106	Hexaconazole	0.01	90.0	4.3	92.1	4.8
			0.02	97.7	3.5	102.8	3.9
			0.1	104.8	4.4	104.9	3.0
84	107	Imazalil	0.01	77.2	11.0	73.5	13.6
			0.02	95.1	6.1	97.6	5.7
			0.1	104.5	1.4	105.7	3.3
85	108	Inpyrfluxam	0.01	91.6	3.6	88.3	2.5
			0.02	98.9	1.9	95.1	3.4
			0.1	101.5	3.5	101.3	2.2
86	109	Ipfencarbazone	0.01	99.3	12.0	113.7	5.1
			0.02	104.9	6.8	114.9	2.5

			0.1	95.9	8.0	108.9	3.5
87	110	Ipflufenoquin	0.01	70.4	10.3	64.4	6.7
			0.02	94.4	7.6	100.9	12.3
			0.1	103.4	9.3	112.5	2.0
88	111	Iprobenfos	0.01	91.4	4.7	92.7	8.0
			0.02	98.6	5.4	106.9	8.5
			0.1	103.0	3.5	109.1	4.4
89	112	Isoprothiolane	0.01	82.9	6.0	80.4	5.9
			0.02	104.8	7.5	93.2	8.0
			0.1	109.2	3.0	100.1	5.1
90	113	Isoxathion	0.01	64.7	3.5	78.0	5.6
			0.02	101.6	9.6	100.1	6.4
			0.1	116.0	4.5	111.2	4.0
91	114	Lufenuron	0.01	99.4	5.0	95.9	8.2
			0.02	102.5	2.8	105.8	4.3
			0.1	102.0	7.5	105.1	7.1
92	115	Malathion	0.01	104.1	13.0	102.7	10.6
			0.02	99.8	5.8	97.0	9.5
			0.1	96.1	4.7	102.6	4.1
93	116	Mefenacet	0.01	94.8	10.1	85.4	7.0
			0.02	101.7	4.9	97.5	7.0
			0.1	105.6	3.0	106.3	5.1
94	117	Metaflumizone (*E*)	0.01	70.2	4.5	97.7	7.4
			0.02	94.5	5.7	103.6	1.8
			0.1	108.0	3.5	107.6	3.9
	118	Metaflumizone (*Z*)	0.01	96.8	6.3	102.7	4.4
			0.02	96.8	5.5	96.9	6.6
			0.1	99.2	1.0	97.8	2.9
95	119	Metalaxyl	0.01	81.1	24.8	88.2	18.3
			0.02	96.5	11.3	95.3	18.1
			0.1	95.6	7.9	106.6	10.1
96	120	Methamidophos	0.01	87.0	7.2	84.8	4.7
			0.02	98.7	2.9	102.1	5.5
			0.1	103.0	3.6	104.1	3.3
97	121	Methidathion	0.01	112.9	4.5	81.6	3.8
			0.02	107.9	5.7	99.6	5.8
			0.1	95.1	2.6	102.4	6.5
98	122	Methiocarb	0.01	81.2	7.3	86.8	8.0
			0.02	99.4	7.4	111.1	4.2
			0.1	105.8	2.7	106.2	5.6
99	123	Methoxyfenozide	0.01	77.5	9.2	82.1	5.8
			0.02	93.7	6.9	95.0	9.7
			0.1	104.5	2.4	102.9	3.8
100	124	Metominostrobin	0.01	70.6	7.1	80.0	5.3
			0.02	97.8	1.8	101.1	9.5
			0.1	113.8	3.3	108.8	3.1
101	125	Metrafenone	0.01	105.6	1.9	105.1	3.3
			0.02	105.5	3.6	100.9	3.3
			0.1	105.8	1.1	99.5	4.0
102	126	Metyltetraprole	0.01	103.4	5.2	104.3	5.2
			0.02	101.2	4.3	106.4	4.2
			0.1	99.4	2.6	103.0	3.3
103	127	Molinate	0.01	93.0	4.7	81.9	12.7
			0.02	97.5	2.4	100.3	6.3
			0.1	102.8	5.6	103.2	2.8
104	128	Monocrotophos	0.01	96.8	16.7	101.7	15.5
			0.02	104.6	7.5	111.0	4.8
			0.1	99.6	5.6	97.5	8.2
105	129	Myclobutanil	0.01	63.4	2.5	68.1	2.6
			0.02	96.9	9.6	93.0	9.9
			0.1	114.9	4.9	111.7	6.0
106	130	Novaluron	0.01	72.5	12.5	82.9	10.7
			0.02	86.3	11.5	91.8	11.5
			0.1	104.6	6.8	96.3	6.8
107	131	Orysastrobin	0.01	97.2	3.9	101.8	5.0
			0.02	103.7	4.0	100.6	6.5

			0.1	100.4	6.0	101.3	3.6
108	132	Oxadiargyl	0.01	101.3	7.9	95.0	8.9
			0.02	105.5	11.2	102.6	4.5
			0.1	100.4	7.8	105.2	2.6
109	133	Oxathiapiprolin	0.01	64.6	3.5	70.6	10.2
			0.02	99.1	9.1	105.3	7.4
			0.1	113.7	3.7	109.4	4.2
110	134	Oxaziclomefone	0.01	82.6	7.0	103.5	6.7
			0.02	96.1	9.3	105.3	7.1
			0.1	102.6	4.3	108.3	8.9
111	135	Oxydemeton-methyl	0.01	102.4	8.2	113.5	4.2
			0.02	109.8	2.6	101.3	7.9
			0.1	102.5	9.0	95.9	9.1
112	136	Penconazole	0.01	86.3	4.6	92.9	6.3
			0.02	109.2	4.8	105.5	5.1
			0.1	105.7	6.5	106.2	3.7
113	137	Pencycuron	0.01	89.7	8.1	99.6	7.5
			0.02	99.8	6.4	102.1	5.7
			0.1	102.8	3.6	104.0	5.3
114	138	Pendimethalin	0.01	103.3	5.6	112.3	4.4
			0.02	105.5	4.6	103.1	4.7
			0.1	98.8	3.9	98.3	3.8
115	139	Penoxsulam	0.01	-	-	99.3	6.4
			0.02	-	-	104.6	8.0
			0.1	-	-	105.4	9.5
116	140	Phorate	0.01	100.2	2.6	88.1	5.0
			0.02	101.3	4.6	97.0	5.9
			0.1	101.1	2.2	102.3	2.0
	141	Phorate oxon sulfone	0.01	79.2	6.2	77.3	2.9
			0.02	101.1	2.4	103.6	4.0
			0.1	105.0	2.6	110.6	3.0
	142	Phorate oxon sulfoxide	0.01	86.0	4.4	83.8	4.6
			0.02	98.4	3.7	102.0	4.8
			0.1	103.8	3.0	106.8	1.9
	143	Phorate oxon	0.01	80.9	10.8	79.4	10.1
			0.02	102.4	2.4	94.3	4.1
			0.1	104.6	6.5	106.2	9.3
	144	Phorate sulfone	0.01	96.8	8.0	69.1	9.2
			0.02	110.9	7.4	99.1	3.5
			0.1	110.0	3.5	109.2	3.5
	145	Phorate sulfoxide	0.01	89.5	1.7	77.6	4.3
			0.02	98.8	1.7	94.8	1.2
			0.1	100.7	1.3	104.6	3.5
117	146	Phoxim	0.01	76.7	7.9	72.9	9.0
			0.02	95.8	5.2	97.4	5.3
			0.1	107.4	2.5	106.6	4.9
118	147	Pirimicarb	0.01	98.6	3.3	80.4	3.6
			0.02	98.5	4.9	107.0	3.1
			0.1	105.1	3.9	105.9	2.0
119	148	Probenazole	0.01	86.1	6.9	80.4	9.1
			0.02	103.7	5.9	107.0	3.1
			0.1	107.0	3.8	105.9	3.7
120	149	Profenofos	0.01	107.5	5.6	80.4	6.4
			0.02	105.4	4.1	107.0	8.7
			0.1	101.9	5.2	105.9	6.5
121	150	Propamocarb	0.01	90.9	5.8	80.4	5.2
			0.02	102.4	3.7	107.0	2.4
			0.1	100.6	2.0	105.9	2.0
122	151	Propiconazole	0.01	93.0	11.5	80.4	4.9
			0.02	101.7	4.1	107.0	4.1
			0.1	100.1	7.5	105.9	3.9
123	152	Propoxur	0.01	94.5	1.9	80.4	6.5
			0.02	106.3	5.6	107.0	8.6
			0.1	107.8	1.9	105.9	3.9
124	153	Propyrisulfuron	0.01	66.8	7.0	80.4	7.6
			0.02	99.6	10.2	107.0	6.5

			0.1	113.0	2.9	105.9	4.4
125	154	Prosulfocarb	0.01	102.1	5.9	89.6	5.7
			0.02	98.9	6.9	95.5	3.1
			0.1	102.8	3.4	100.1	5.2
126	155	Pydiflumetofen	0.01	63.1	4.4	79.8	17.2
			0.02	94.3	3.9	99.6	13.3
			0.1	113.0	6.6	109.7	11.4
127	156	Pyraclostrobin	0.01	90.8	12.7	95.9	7.1
			0.02	99.8	8.2	103.5	5.2
			0.1	101.4	2.5	103.0	5.9
128	157	Pyribencarb	0.01	79.9	7.5	81.8	7.0
			0.02	97.3	4.5	97.6	7.2
			0.1	105.1	1.7	108.4	3.1
129	158	Pyributicarb	0.01	91.8	7.5	93.2	10.1
			0.02	102.3	6.2	105.3	2.1
			0.1	104.8	7.4	103.7	4.4
130	159	Pyridaben	0.01	106.0	2.9	100.9	1.7
			0.02	102.7	0.9	101.1	1.9
			0.1	100.6	0.8	100.0	1.3
131	160	Pyriofenone	0.01	84.2	13.5	92.5	10.7
			0.02	98.7	8.5	102.8	10.7
			0.1	104.5	8.2	113.4	3.4
132	161	Pyriproxyfen	0.01	93.5	5.2	89.5	4.7
			0.02	98.4	2.5	100.7	1.7
			0.1	98.5	2.0	102.5	3.2
133	162	Pyroquilon	0.01	89.1	6.1	87.7	8.1
			0.02	95.8	6.5	97.6	6.1
			0.1	100.8	4.1	104.5	4.5
134	163	Quinoclamine	0.01	100.9	5.1	98.4	7.4
			0.02	98.8	6.4	99.7	5.9
			0.1	95.6	1.8	95.7	4.8
135	164	Saflufenacil	0.01	94.9	7.7	101.5	7.8
			0.02	103.8	6.7	104.4	6.9
			0.1	103.8	3.9	106.0	2.8
136	165	Sedaxane	0.01	71.3	17.1	63.1	3.3
			0.02	97.7	14.6	87.9	15.8
			0.1	106.3	9.2	109.2	6.7
137	166	Sethoxydim	0.01	86.0	2.5	97.5	3.6
			0.02	93.0	7.4	100.0	1.7
			0.1	98.3	1.4	101.1	2.9
138	167	Simazine	0.01	81.8	9.7	82.5	15.2
			0.02	95.2	11.4	101.9	5.1
			0.1	97.8	3.0	108.2	8.5
	168	Shimazine-2-hydroxy (OH-Simazine)	0.01	-	-	-	-
			0.02	-	-	-	-
			0.1	-	-	-	-
139	169	Spinetoram J	0.01	101.9	5.7	92.1	13.3
			0.02	99.5	8.5	100.6	10.6
			0.1	103.9	3.7	100.0	6.5
	170	Spinetoram L	0.01	106.2	1.3	100.3	1.5
			0.02	102.6	3.1	98.7	2.4
			0.1	102.9	2.0	100.6	2.5
140	171	Spinosyn A	0.01	96.3	5.5	105.3	2.2
			0.02	99.0	3.2	102.6	3.7
			0.1	101.1	6.0	100.1	3.5
	172	Spinosyn D	0.01	100.5	4.2	103.4	4.3
			0.02	102.6	1.9	101.3	2.7
			0.1	101.4	2.4	100.4	4.7
141	173	Spiromesifen	0.01	73.6	13.1	90.4	10.5
			0.02	99.1	8.5	108.7	7.6
			0.1	102.8	7.3	102.0	7.4
142	174	Spirotetramat	0.01	86.1	4.7	85.1	5.9
			0.02	98.7	3.6	101.8	2.9
			0.1	104.8	3.6	101.5	4.2
143	175	Sulfosulfuro	0.01	83.1	7.7	87.2	7.0
			0.02	92.7	7.0	105.6	8.6

			0.1	105.2	3.9	109.4	4.4
144	176	Sulfoxaflor	0.01	74.2	8.1	75.3	2.0
			0.02	99.0	3.1	100.1	2.6
			0.1	108.0	0.3	106.6	2.8
145	177	Tebuconazole	0.01	105.4	8.2	104.5	6.9
			0.02	102.1	5.6	109.7	5.8
			0.1	99.9	4.5	100.0	6.6
146	178	Tebufenozide	0.01	85.4	7.4	105.0	8.0
			0.02	100.2	10.5	102.2	8.5
			0.1	107.0	3.6	99.3	5.5
147	179	Tebufloquin	0.01	80.8	5.3	75.3	9.7
			0.02	97.1	6.6	99.4	3.3
			0.1	109.2	4.6	105.5	2.9
	180	Tebufloquin M1	0.01	93.3	10.2	94.8	8.1
			0.02	100.1	5.0	110.7	4.4
			0.1	106.9	4.1	107.7	3.6
148	181	Teflubenzuron	0.01	105.8	4.5	89.0	10.0
			0.02	99.7	6.2	91.7	1.8
			0.1	100.8	2.3	103.1	4.8
149	182	Terbuthylazine	0.01	78.7	15.0	80.8	9.7
			0.02	102.8	5.7	97.2	5.7
			0.1	109.4	2.7	108.0	9.2
	183	Terbuthylazine-2-hydroxy (OH-TER)	0.01	-	-	-	-
			0.02	-	-	-	-
			0.1	-	-	-	-
	184	Terbuthylazine-desethyl (DE-TER)	0.01	82.9	4.3	69.2	7.1
			0.02	100.1	3.0	103.7	4.1
			0.1	107.6	2.6	110.0	3.3
	185	Terbuthylazine-desethyl-2-hydroxy (OH-DE-TER)	0.01	-	-	-	-
			0.02	-	-	-	-
			0.1	-	-	-	-
150	186	Tetraniliprole	0.01	94.1	18.0	100.2	14.5
			0.02	85.0	11.3	108.4	7.0
			0.1	103.1	5.8	102.2	5.7
151	187	Thiabendazole	0.01	97.9	6.0	107.8	2.7
			0.02	107.5	11.2	107.6	9.2
			0.1	103.2	4.7	95.2	5.5
	188	5-Hydroxy thiabendazole	0.01	92.9	1.6	79.3	9.5
			0.02	99.4	3.0	103.9	5.6
			0.1	104.3	5.2	106.1	14.9
152	189	Thiacloprid	0.01	82.9	6.8	86.4	4.9
			0.02	102.3	2.7	103.1	1.8
			0.1	102.4	4.3	106.0	3.0
153	190	Thiamethoxam	0.01	102.9	2.0	81.3	2.5
			0.02	107.3	5.1	98.7	1.5
			0.1	106.4	5.0	106.0	1.5
154	191	Thiobencarb	0.01	90.9	3.8	78.6	7.3
			0.02	105.7	7.7	101.5	4.2
			0.1	101.1	5.6	106.1	3.2
155	192	Tiadinil	0.01	113.6	4.8	112.3	6.6
			0.02	103.8	5.7	104.6	8.8
			0.1	98.9	3.5	99.0	1.9
156	193	Tolprocarb	0.01	88.4	5.7	67.7	6.3
			0.02	100.8	4.0	95.5	1.7
			0.1	102.3	3.7	107.7	4.6
157	194	Trichlorfon(Metrifonate)	0.01	78.0	12.8	89.4	9.5
			0.02	81.4	17.8	76.2	5.5
			0.1	98.1	4.3	108.2	2.2
158	195	Tricyclazole	0.01	87.9	3.5	92.5	1.3
			0.02	98.3	2.8	101.2	2.3
			0.1	101.1	1.3	102.9	2.0
159	196	Trifloxystrobin	0.01	85.5	6.0	105.9	4.6
			0.02	93.4	6.0	101.8	5.3
			0.1	100.9	5.2	103.0	4.8
160	197	Triflumezopyrim	0.01	95.2	6.2	86.2	4.9
			0.02	103.2	3.1	102.4	5.2

			0.1	103.2	5.2	100.2	2.5
161	198	Warfarin	0.01	93.1	5.7	81.5	8.0
			0.02	105.1	2.5	99.7	3.6
			0.1	106.3	4.7	107.2	7.2

Table S5. Assessment of dietary exposure to chemicals in fishery products based on average consumption

Chemical	Fishery product	Group	Intake rate (kg/day)	Maximum concentration (mg/kg)	Body weight (kg)	Estimated daily intake (mg/kg·BW/day)	Acceptable daily intake (mg/kg·BW/day)	%ADI
Ethoxyquin	Croaker	All ages	0.0020	0.970	59.71	3.19×10^{-5}	0.005	0.6
		1-2	0.0028		12.60	2.14×10^{-4}		4.3
		3-6	0.0035		19.61	1.72×10^{-4}		3.4
		7-12	0.0011		38.40	2.71×10^{-5}		0.5
		13-19	0.0015		61.66	2.31×10^{-5}		0.5
		20-64	0.0018		65.96	2.71×10^{-5}		0.5
		65 older	0.0023		60.39	3.71×10^{-5}		0.7
		20 under	0.0018		40.01	4.40×10^{-5}		0.9
		20 older	0.0020		64.27	3.02×10^{-5}		0.6
		Male	0.0023		65.60	3.41×10^{-5}		0.7
		Female	0.0017		54.99	2.99×10^{-5}		0.6
Ethoxyquin	Sea bass	All ages	0.0000	0.015	59.71	6.58×10^{-9}	0.005	0.0
		1-2	-		12.60	-		-
		3-6	-		19.61	-		-
		7-12	-		38.40	-		-
		13-19	-		61.66	-		-
		20-64	0.0000		65.96	9.84×10^{-9}		0.0
		65 older	0.0000		60.39	1.59×10^{-9}		0.0
		20 under	-		40.01	-		-
		20 older	0.0000		64.27	7.53×10^{-9}		0.0
		Male	0.0001		65.60	1.28×10^{-8}		0.0
		Female	0.0000		54.99	6.57×10^{-10}		0.0
Ethoxyquin	Flatfish	All ages	0.0010	0.010	59.71	1.63×10^{-7}	0.005	0.0
		1-2	-		12.60	-		-
		3-6	0.0002		19.61	8.79×10^{-8}		0.0
		7-12	0.0006		38.40	1.44×10^{-7}		0.0
		13-19	0.0011		61.66	1.85×10^{-7}		0.0
		20-64	0.0012		65.96	1.83×10^{-7}		0.0
		65 older	0.0007		60.39	1.19×10^{-7}		0.0
		20 under	0.0006		40.01	1.45×10^{-7}		0.0
		20 older	0.0011		64.27	1.66×10^{-7}		0.0
		Male	0.0013		65.60	1.96×10^{-7}		0.0
		Female	0.0007		54.99	1.31×10^{-7}		0.0

Table S5. Countinued

Chemical	Fishery product	Group	Intake rate (kg/day)	Maximum concentration (mg/kg)	Body weight (kg)	Estimated daily intake (mg/kg·BW/day)	Acceptable daily intake (mg/kg·BW/day)	%ADI
Ethoxyquin	Flounder	All ages	0.0001	0.015	59.71	2.51×10^{-8}	0.005	0.0
		1-2	-		12.60	-		-
		3-6	-		19.61	-		-
		7-12	-		38.40	-		-
		13-19	-		61.66	-		-
		20-64	0.0001		65.96	1.99×10^{-8}		0.0
		65 older	0.0002		60.39	4.97×10^{-8}		0.0
		20 under	-		40.01	-		-
		20 older	0.0001		64.27	2.87×10^{-8}		0.0
		Male	0.0001		65.60	3.29×10^{-8}		0.0
		Female	0.0001		54.99	1.75×10^{-8}		0.0
Ethoxyquin	Snapper	All ages	0.0004	0.025	59.71	1.73×10^{-7}	0.005	0.0
		1-2	0.0001		12.60	2.17×10^{-7}		0.0
		3-6	0.0003		19.61	4.36×10^{-7}		0.0
		7-12	0.0002		38.40	9.79×10^{-8}		0.0
		13-19	-		61.66	-		-
		20-64	0.0004		65.96	1.47×10^{-7}		0.0
		65 older	0.0007		60.39	2.76×10^{-7}		0.0
		20 under	0.0001		40.01	8.70×10^{-8}		0.0
		20 older	0.0005		64.27	1.85×10^{-7}		0.0
		Male	0.0005		65.60	1.91×10^{-7}		0.0
		Female	0.0003		54.99	1.56×10^{-7}		0.0
Ethoxyquin	Yellow tail	All ages	0.0001	0.035	59.71	8.63×10^{-8}	0.005	0.0
		1-2	-		12.60	-		-
		3-6	0.0000		19.61	4.19×10^{-8}		0.0
		7-12	0.0001		38.40	8.73×10^{-8}		0.0
		13-19	0.0002		61.66	1.15×10^{-7}		0.0
		20-64	0.0002		65.96	7.97×10^{-8}		0.0
		65 older	0.0002		60.39	9.97×10^{-8}		0.0
		20 under	0.0001		40.01	8.79×10^{-8}		0.0
		20 older	0.0002		64.27	8.61×10^{-8}		0.0
		Male	0.0002		65.60	1.27×10^{-7}		0.0
		Female	0.0001		54.99	4.77×10^{-8}		0.0

Table S5. Countinued

Chemical	Fishery product	Group	Intake rate (kg/day)	Maximum concentration (mg/kg)	Body weight (kg)	Estimated daily intake (mg/kg·BW/day)	Acceptable daily intake (mg/kg·BW/day)	%ADI
Ethoxyquin	Loach	All ages	0.0009	0.140	59.71	2.16×10^{-6}	0.005	0.0
		1-2	-		12.60	-		-
		3-6	0.0000		19.61	1.75×10^{-7}		0.0
		7-12	0.0001		38.40	5.21×10^{-7}		0.0
		13-19	0.0001		61.66	3.37×10^{-7}		0.0
		20-64	0.0009		65.96	1.87×10^{-6}		0.0
		65 older	0.0016		60.39	3.71×10^{-6}		0.1
		20 under	0.0001		40.01	3.59×10^{-7}		0.0
		20 older	0.0011		64.27	2.42×10^{-6}		0.0
		Male	0.0011		65.60	2.43×10^{-6}		0.0
		Female	0.0007		54.99	1.91×10^{-6}		0.0
Ethoxyquin	Eel	All ages	0.0013	0.445	59.71	9.37×10^{-6}	0.005	0.2
		1-2	-		12.60	-		-
		3-6	0.0001		19.61	1.73×10^{-6}		0.0
		7-12	0.0003		38.40	3.01×10^{-6}		0.1
		13-19	0.0005		61.66	3.47×10^{-6}		0.1
		20-64	0.0013		65.96	8.59×10^{-6}		0.2
		65 older	0.0019		60.39	1.43×10^{-5}		0.3
		20 under	0.0003		40.01	3.16×10^{-6}		0.1
		20 older	0.0015		64.27	1.03×10^{-5}		0.2
		Male	0.0015		65.60	1.02×10^{-5}		0.2
		Female	0.0011		54.99	8.55×10^{-6}		0.2
Ethoxyquin	Catfish	All ages	0.0001	0.040	59.71	7.72×10^{-8}	0.005	0.0
		1-2	-		12.60	-		-
		3-6	-		19.61	-		-
		7-12	-		38.40	-		-
		13-19	-		61.66	-		-
		20-64	0.0001		65.96	7.35×10^{-8}		0.0
		65 older	0.0002		60.39	1.23×10^{-7}		0.0
		20 under	-		40.01	-		-
		20 older	0.0001		64.27	8.84×10^{-8}		0.0
		Male	0.0002		65.60	9.21×10^{-8}		0.0
		Female	0.0001		54.99	6.31×10^{-8}		0.0

Table S5. Countinued

Chemical	Fishery product	Group	Intake rate (kg/day)	Maximum concentration (mg/kg)	Body weight (kg)	Estimated daily intake (mg/kg·BW/day)	Acceptable daily intake (mg/kg·BW/day)	%ADI
Ethoxyquin	Leather carp	All ages	0.0000	0.105	59.71	1.36×10^{-8}	0.005	0.0
		1-2	-		12.60	-		-
		3-6	-		19.61	-		-
		7-12	-		38.40	-		-
		13-19	-		61.66	-		-
		20-64	0.0000		65.96	5.43×10^{-9}		0.0
		65 older	0.0000		60.39	4.03×10^{-8}		0.0
		20 under	-		40.01	-		-
		20 older	0.0000		64.27	1.56×10^{-8}		0.0
		Male	-		65.60	-		-
		Female	0.0000		54.99	2.66×10^{-8}		0.0
Ethoxyquin	Carp	All ages	0.0000	0.030	59.71	4.68×10^{-9}	0.005	0.0
		1-2	-		12.60	-		-
		3-6	-		19.61	-		-
		7-12	-		38.40	-		-
		13-19	-		61.66	-		-
		20-64	0.0000		65.96	4.60×10^{-9}		0.0
		65 older	0.0000		60.39	7.07×10^{-9}		0.0
		20 under	-		40.01	-		-
		20 older	0.0000		64.27	5.35×10^{-9}		0.0
		Male	0.0000		65.60	3.66×10^{-9}		0.0
		Female	0.0000		54.99	5.64×10^{-9}		0.0
Pendimethalin	Loach	All ages	0.0009	0.930	59.71	1.44×10^{-5}	0.13	0.0
		1-2	-		12.60	-		-
		3-6	0.0000		19.61	1.16×10^{-6}		0.0
		7-12	0.0001		38.40	3.46×10^{-6}		0.0
		13-19	0.0001		61.66	2.24×10^{-6}		0.0
		20-64	0.0009		65.96	1.24×10^{-5}		0.0
		65 older	0.0016		60.39	2.47×10^{-5}		0.0
		20 under	0.0001		40.01	2.38×10^{-6}		0.0
		20 older	0.0011		64.27	1.61×10^{-5}		0.0
		Male	0.0011		65.60	1.61×10^{-5}		0.0
		Female	0.0007		54.99	1.27×10^{-5}		0.0

Table S5. Countinued

Chemical	Fishery product	Group	Intake rate (kg/day)	Maximum concentration (mg/kg)	Body weight (kg)	Estimated daily intake (mg/kg·BW/day)	Acceptable daily intake (mg/kg·BW/day)	%ADI
Pendimethalin	Catfish	All ages	0.0001	0.020	59.71	3.86×10^{-8}	0.13	0.0
		1-2	-		12.60	-		-
		3-6	-		19.61	-		-
		7-12	-		38.40	-		-
		13-19	-		61.66	-		-
		20-64	0.0001		65.96	3.68×10^{-8}		0.0
		65 older	0.0002		60.39	6.14×10^{-8}		0.0
		20 under	-		40.01	-		-
		20 older	0.0001		64.27	4.42×10^{-8}		0.0
		Male	0.0002		65.60	4.60×10^{-8}		0.0
		Female	0.0001		54.99	3.15×10^{-8}		0.0
Pendimethalin	Crucian carp	All ages	0.0000	0.030	59.71	5.31×10^{-9}	0.13	0.0
		1-2	-		12.60	-		-
		3-6	-		19.61	-		-
		7-12	-		38.40	-		-
		13-19	-		61.66	-		-
		20-64	0.0000		65.96	8.45×10^{-9}		0.0
		65 older	-		60.39	-		-
		20 under	-		40.01	-		-
		20 older	0.0000		64.27	6.07×10^{-9}		0.0
		Male	-		65.60	-		-
		Female	0.0000		54.99	1.04×10^{-8}		0.0
Pendimethalin	Carp	All ages	0.0000	0.010	59.71	1.56×10^{-9}	0.13	0.0
		1-2	-		12.60	-		-
		3-6	-		19.61	-		-
		7-12	-		38.40	-		-
		13-19	-		61.66	-		-
		20-64	0.0000		65.96	1.53×10^{-9}		0.0
		65 older	0.0000		60.39	2.36×10^{-9}		0.0
		20 under	-		40.01	-		-
		20 older	0.0000		64.27	1.78×10^{-9}		0.0
		Male	0.0000		65.60	1.22×10^{-9}		0.0
		Female			54.99	1.88×10^{-9}		0.0

Table S5. Countinued

Chemical	Fishery product	Group	Intake rate (kg/day)	Maximum concentration (mg/kg)	Body weight (kg)	Estimated daily intake (mg/kg·BW/day)	Acceptable daily intake (mg/kg·BW/day)	%ADI
Lufenuron	Salmon	All ages	0.0006	0.040	59.71	3.74×10^{-7}	0.015	0.0
		1-2	0.0001		12.60	2.75×10^{-7}		0.0
		3-6	0.0001		19.61	3.04×10^{-7}		0.0
		7-12	0.0004		38.40	4.48×10^{-7}		0.0
		13-19	0.0006		61.66	4.16×10^{-7}		0.0
		20-64	0.0008		65.96	4.57×10^{-7}		0.0
		65 older	0.0002		60.39	1.51×10^{-7}		0.0
		20 under	0.0004		40.01	3.90×10^{-7}		0.0
		20 older	0.0006		64.27	3.72×10^{-7}		0.0
		Male	0.0005		65.60	3.32×10^{-7}		0.0
		Female	0.0006		54.99	4.14×10^{-7}		0.0
Lufenuron	Eel	All ages	0.0013	1.580	59.71	3.33×10^{-5}	0.015	0.2
		1-2	-		12.60	-		-
		3-6	0.0001		19.61	6.13×10^{-6}		0.0
		7-12	0.0003		38.40	1.07×10^{-5}		0.1
		13-19	0.0005		61.66	1.23×10^{-5}		0.1
		20-64	0.0013		65.96	3.05×10^{-5}		0.2
		65 older	0.0019		60.39	5.07×10^{-5}		0.3
		20 under	0.0003		40.01	1.12×10^{-5}		0.1
		20 older	0.0015		64.27	3.64×10^{-5}		0.2
		Male	0.0015		65.60	3.63×10^{-5}		0.2
		Female	0.0011		54.99	3.04×10^{-5}		0.2
Lufenuron	Catfish	All ages	0.0001	0.010	59.71	1.93×10^{-8}	0.015	0.0
		1-2	-		12.60	-		-
		3-6	-		19.61	-		-
		7-12	-		38.40	-		-
		13-19	-		61.66	-		-
		20-64	0.0001		65.96	1.84×10^{-8}		0.0
		65 older	0.0002		60.39	3.07×10^{-8}		0.0
		20 under	-		40.01	-		-
		20 older	0.0001		64.27	2.21×10^{-8}		0.0
		Male	0.0002		65.60	2.30×10^{-8}		0.0
		Female	0.0001		54.99	1.58×10^{-8}		0.0

Table S5. Countinued

Chemical	Fishery product	Group	Intake rate (kg/day)	Maximum concentration (mg/kg)	Body weight (kg)	Estimated daily intake (mg/kg·BW/day)	Acceptable daily intake (mg/kg·BW/day)	%ADI
Lufenuron	Trout	All ages	0.0001	0.020	59.71	1.99×10^{-8}	0.015	0.0
		1-2	-		12.60	-		-
		3-6	-		19.61	-		-
		7-12	-		38.40	-		-
		13-19	-		61.66	-		-
		20-64	0.0001		65.96	1.65×10^{-8}		0.0
		65 older	0.0001		60.39	3.79×10^{-8}		0.0
		20 under	-		40.01	-		-
		20 older	0.0001		64.27	2.28×10^{-8}		0.0
		Male	0.0001		65.60	4.08×10^{-8}		0.0
		Female	-		54.99	-		-
Phoxim	Loach	All ages	0.0009	1.880	59.71	2.90×10^{-5}	0.004	0.7
		1-2	-		12.60	-		-
		3-6	0.0000		19.61	2.34×10^{-6}		0.1
		7-12	0.0001		38.40	7.00×10^{-6}		0.2
		13-19	0.0001		61.66	4.52×10^{-6}		0.1
		20-64	0.0009		65.96	2.51×10^{-5}		0.6
		65 older	0.0016		60.39	4.99×10^{-5}		1.2
		20 under	0.0001		40.01	4.82×10^{-6}		0.1
		20 older	0.0011		64.27	3.25×10^{-5}		0.8
		Male	0.0011		65.60	3.26×10^{-5}		0.8
		Female	0.0007		54.99	2.56×10^{-5}		0.6
Phoxim	Eel	All ages	0.0013	0.020	59.71	4.21×10^{-7}	0.004	0.0
		1-2	-		12.60	-		-
		3-6	0.0001		19.61	7.76×10^{-8}		0.0
		7-12	0.0003		38.40	1.35×10^{-7}		0.0
		13-19	0.0005		61.66	1.56×10^{-7}		0.0
		20-64	0.0013		65.96	3.86×10^{-7}		0.0
		65 older	0.0019		60.39	6.42×10^{-7}		0.0
		20 under	0.0003		40.01	1.42×10^{-7}		0.0
		20 older	0.0015		64.27	4.61×10^{-7}		0.0
		Male	0.0015		65.60	4.59×10^{-7}		0.0
		Female	0.0011		54.99	3.84×10^{-7}		0.0

Table S5. Countinued

Chemical	Fishery product	Group	Intake rate (kg/day)	Maximum concentration (mg/kg)	Body weight (kg)	Estimated daily intake (mg/kg·BW/day)	Acceptable daily intake (mg/kg·BW/day)	%ADI
Trichlorfon	Loach	All ages	0.0009	0.420	59.71	6.48×10^{-6}	0.002	0.3
		1-2	-		12.60	-		-
		3-6	0.0000		19.61	5.24×10^{-7}		0.0
		7-12	0.0001		38.40	1.56×10^{-6}		0.1
		13-19	0.0001		61.66	1.01×10^{-6}		0.1
		20-64	0.0009		65.96	5.61×10^{-6}		0.3
		65 older	0.0016		60.39	1.11×10^{-5}		0.6
		20 under	0.0001		40.01	1.08×10^{-6}		0.1
		20 older	0.0011		64.27	7.26×10^{-6}		0.4
		Male	0.0011		65.60	7.28×10^{-6}		0.4
		Female	0.0007		54.99	5.72×10^{-6}		0.3
Oxadiargyl	Catfish	All ages	0.0001	0.040	59.71	7.72×10^{-8}	0.008	0.0
		1-2	-		12.60	-		-
		3-6	-		19.61	-		-
		7-12	-		38.40	-		-
		13-19	-		61.66	-		-
		20-64	0.0001		65.96	7.35×10^{-8}		0.0
		65 older	0.0002		60.39	1.23×10^{-7}		0.0
		20 under	-		40.01	-		-
		20 older	0.0001		64.27	8.84×10^{-8}		0.0
		Male	0.0002		65.60	9.21×10^{-8}		0.0
		Female	0.0001		54.99	6.31×10^{-8}		0.0
Metaflumizone	Gizzard shad	All ages	0.0002	0.030	59.71	7.80×10^{-8}	0.01	0.0
		1-2	-		12.60	-		-
		3-6	-		19.61	-		-
		7-12	-		38.40	-		-
		13-19	0.0000		61.66	3.92×10^{-9}		0.0
		20-64	0.0002		65.96	9.62×10^{-8}		0.0
		65 older	0.0001		60.39	6.86×10^{-8}		0.0
		20 under	0.0000		40.01	1.77×10^{-9}		0.0
		20 older	0.0002		64.27	8.90×10^{-8}		0.0
		Male	0.0002		65.60	9.92×10^{-8}		0.0
		Female	0.0001		54.99	5.77×10^{-8}		0.0

Table S5. Countinued

Chemical	Fishery product	Group	Intake rate (kg/day)	Maximum concentration (mg/kg)	Body weight (kg)	Estimated daily intake (mg/kg · BW/day)	Acceptable daily intake (mg/kg · BW/day)	%ADI
Metaflumizone	Loach	All ages	0.0009	0.010	59.71	1.54×10^{-7}	0.01	0.0
		1-2	-		12.60	-		-
		3-6	0.0000		19.61	1.25×10^{-8}		0.0
		7-12	0.0001		38.40	3.72×10^{-8}		0.0
		13-19	0.0001		61.66	2.41×10^{-8}		0.0
		20-64	0.0009		65.96	1.34×10^{-7}		0.0
		65 older	0.0016		60.39	2.65×10^{-7}		0.0
		20 under	0.0001		40.01	2.56×10^{-8}		0.0
		20 older	0.0011		64.27	1.73×10^{-7}		0.0
		Male	0.0011		65.60	1.73×10^{-7}		0.0
		Female	0.0007		54.99	1.36×10^{-7}		0.0
Propiconazole	Flounder	All ages	0.0001	0.010	59.71	1.67×10^{-8}	0.07	0.0
		1-2	-		12.60	-		-
		3-6	-		19.61	-		-
		7-12	-		38.40	-		-
		13-19	-		61.66	-		-
		20-64	0.0001		65.96	1.32×10^{-8}		0.0
		65 older	0.0002		60.39	3.32×10^{-8}		0.0
		20 under	-		40.01	-		-
		20 older	0.0001		64.27	1.91×10^{-8}		0.0
		Male	0.0001		65.60	2.20×10^{-8}		0.0
		Female	0.0001		54.99	1.17×10^{-8}		0.0
Ipfencarbazone	Trout	All ages	0.0001	0.010	59.71	9.97×10^{-9}	0.001	0.0
		1-2	-		12.60	-		-
		3-6	-		19.61	-		-
		7-12	-		38.40	-		-
		13-19	-		61.66	-		-
		20-64	0.0001		65.96	8.25×10^{-9}		0.0
		65 older	0.0001		60.39	1.89×10^{-8}		0.0
		20 under	-		40.01	-		-
		20 older	0.0001		64.27	1.14×10^{-8}		0.0
		Male	0.0001		65.60	2.04×10^{-8}		0.0
		Female	-		54.99	-		-

Table S5. Countinued

Chemical	Fishery product	Group	Intake rate (kg/day)	Maximum concentration (mg/kg)	Body weight (kg)	Estimated daily intake (mg/kg·BW/day)	Acceptable daily intake (mg/kg·BW/day)	%ADI
Isoprothiolane	Catfish	All ages	0.0001	0.010	59.71	1.93×10^{-8}	0.1	0.0
		1-2	-		12.60	-		-
		3-6	-		19.61	-		-
		7-12	-		38.40	-		-
		13-19	-		61.66	-		-
		20-64	0.0001		65.96	1.84×10^{-8}		0.0
		65 older	0.0002		60.39	3.07×10^{-8}		0.0
		20 under	-		40.01	-		-
		20 older	0.0001		64.27	2.21×10^{-8}		0.0
		Male	0.0002		65.60	2.30×10^{-8}		0.0
		Female	0.0001		54.99	1.58×10^{-8}		0.0

Table S6. Acceptable daily intake (ADI) values for all analyzed compounds

No.	Pesticide	Acceptable daily intake (mg/kg・BW /day)	Reference
1	Acephate	0.03	MFDS
2	Acetamiprid	0.071	MFDS
3	Acynonapyr	0.04	MFDS
4	Alachlor	0.01	MFDS
5	Aldicarb	0.003	MFDS
6	Amitraz	0.01	MFDS
7	Atrazine	0.02	EFSA, WHO
8	Azinphos-methyl	0.03	WHO
9	Azoxystrobin	0.2	MFDS
10	Bendiocarb	0.004	MFDS
11	Bensulfuron methyl	0.2	EFSA
12	Benzovindiflupyr	0.049	MFDS
13	Benzpyrimoxan	0.1	WHO
14	Bifenazate	0.01	MFDS
15	Bioresmethrin	0.03	MFDS
16	Boscalid	0.04	MFDS
17	Brodifacoum	-	-
18	Buprofezin	0.01	MFDS
19	Butamifos	0.008	FSCJ
20	Cafenstrole	0.003	MFDS
21	Carbaryl	0.0075	MFDS
22	Carbendazim	0.03	MFDS
23	Carbofuran	0.001	MFDS
24	Carbosulfan	0.001	MFDS
25	Carfentrazone-ethyl	0.03	MFDS
26	Carpropamide	0.014	MFDS
27	Chlorantraniliprole	2	MFDS
28	Chlorfenvinphos	0.0005	MFDS
29	Chromafenozide	0.27	MFDS
30	Clofentezine	0.017	MFDS
31	Clomeprop	0.0062	RDA
32	Clothianidin	0.097	MFDS
33	Cumyluron	-	-
34	Cyantraniliprole	0.057	MFDS
35	Cyclopyrimorate	0.063	FSCJ
36	Cyproconazole	0.02	MFDS
37	Daimuron	0.3	MFDS
38	Diazinon	0.0002	MFDS
39	Dichlorvos (DDVP)	0.004	MFDS

40	Diclocymet	-	-
41	Diflubenzuron	0.02	MFDS
42	Dimethomorph	0.2	MFDS
43	Dinotefuran	0.02	MFDS
44	Disulfoton	0.00004	MFDS
45	Diuron	0.007	MFDS
46	Edifenphos	0.003	MFDS
47	Emamectin benzoate	0.0025	MFDS
48	Epoxiconazole	0.007	MFDS
49	Esprocarb	0.01	MFDS
50	Ethiofencarb	0.1	MFDS
51	Ethiprole	0.005	MFDS
52	Ethoxyquin	0.005	MFDS
53	Etobenzanid	-	-
54	Etoxazole	0.04	MFDS
55	Etrimfos	0.003	MFDS
56	Famoxadone	0.006	MFDS
57	Fenamidone	0.028	MFDS
58	Fenarimol	0.01	MFDS
59	Fenbuconazole	0.03	MFDS
60	Fenhexamid	0.2	MFDS
61	Fenobucarb	0.014	MFDS
62	Fenoxasulfone	0.018	MFDS
63	Fenpyroximate	0.01	MFDS
64	Fensulfothion	0.0003	MFDS
65	Fenthion (MPP)	0.007	WHO
66	Fentrazamide	0.0052	MFDS
67	Ferimzone	0.019	MFDS
68	Flonicamid	0.025	MFDS
69	Florpyrauxifen-benzyl	2.4	MFDS
70	Fluazinam	0.01	MFDS
71	Flubendiamide	0.017	MFDS
72	Fludioxonil	0.4	MFDS
73	Flufenoxuron	0.037	MFDS
74	Flumioxazin	0.02	WHO
75	Fluopicolide	0.079	MFDS
76	Flupyrimin	0.01	MFDS
77	Fluralaner	0.01	MFDS
78	Fluridone	0.08	EPA
79	Flutolanil	0.09	MFDS
80	Fluxametamide	0.0085	MFDS
81	Fluxapyroxad	0.021	MFDS

82	Furametpyr	-	-
83	Hexaconazole	0.005	MFDS
84	Imazalil	0.03	MFDS
82	Inpyrfluxam	0.06	WHO
86	Ipfencarbazone	0.001	MFDS
87	Ipflufenoquin	0.05	MFDS
88	Iprobenfos	0.035	MFDS
89	Isoprothiolane	0.1	MFDS
90	Isoxathion	0.02	FSCJ
91	Lufenuron	0.015	MFDS
92	Malathion	0.029	MFDS
93	Mefenacet	0.007	MFDS
94	Metaflumizone	0.1	MFDS
95	Metalaxyl	0.08	MFDS
96	Methamidophos	0.004	MFDS
97	Methidathion	0.001	MFDS
98	Methiocarb	0.02	MFDS
99	Methoxyfenozide	0.1	MFDS
100	Metominostrobin	0.016	FSCJ
101	Metrafenone	0.25	MFDS
102	Metyltetraprole	2.5	FSCJ
103	Molinate	0.0021	MFDS
104	Monocrotophos	0.0006	MFDS
105	Myclobutanil	0.03	MFDS
106	Novaluron	0.01	MFDS
107	Orysastrobin	0.052	MFDS
108	Oxadiargyl	0.008	MFDS
109	Oxathiapiprolin	1.04	MFDS
110	Oxaziclomefone	0.0091	MFDS
111	Oxydemeton-methyl	0.0003	EFSA
112	Penconazole	0.03	MFDS
113	Pencycuron	0.2	MFDS
114	Pendimethalin	0.13	MFDS
115	Penoxsulam	0.05	MFDS
116	Phorate	0.0007	MFDS
117	Phoxim	0.004	MFDS
118	Pirimicarb	0.02	MFDS
119	Probenazole	0.01	MFDS
120	Profenofos	0.03	MFDS
121	Propamocarb	0.4	MFDS
122	Propiconazole	0.07	MFDS
123	Propoxur	0.005	MFDS

124	Propyrisulfuron	0.011	MFDS
125	Prosulfocarb	0.005	EFSA
126	Pydiflumetofen	0.092	MFDS
127	Pyraclostrobin	0.03	MFDS
128	Pyribencarb	0.039	MFDS
129	Pyributicarb	0.0088	MFDS
130	Pyridaben	0.005	MFDS
131	Pyriofenone	0.091	MFDS
132	Pyriproxyfen	0.1	MFDS
133	Pyroquilon	0.019	FSCJ
134	Quinoclamine	0.0021	MFDS
135	Saflufenacil	0.046	MFDS
136	Sedaxane	0.11	MFDS
137	Sethoxydim	0.14	MFDS
138	Simazine	0.018	MFDS
139	Spinetoram	0.05	MFDS
140	Spinosyn A	0.02	MFDS
141	Spiromesifen	0.03	MFDS
142	Spirotetramat	0.05	MFDS
143	Sulfosulfuron	0.24	EFSA
144	Sulfoxaflor	0.05	MFDS
145	Tebuconazole	0.03	MFDS
146	Tebufenozide	0.02	MFDS
147	Tebufloquin	0.041	MFDS
148	Teflubenzuron	0.01	MFDS
149	Terbuthylazine	0.004	EFSA
150	Tetraniliprole	0.88	MFDS
151	Thiabendazole	0.1	MFDS
152	Thiacloprid	0.01	MFDS
153	Thiamethoxam	0.08	MFDS
154	Thiobencarb	0.009	MFDS
155	Tiadinil	0.04	MFDS
156	Tolprocarb	-	-
157	Trichlorfon (Metrifonate)	0.002	WHO
158	Tricyclazole	0.05	MFDS
159	Trifloxystrobin	0.04	MFDS
160	Triflumezopyrim	0.12	MFDS
161	Warfarin	0.0003	EPA

Reference

Codex Alimentarius Commission. Guidelines on Good Laboratory Practice in Pesticide Residue Analysis (CAC/GL 40-1993). Available online: https://www.fao.org/input/download/standards/378/cxg_040e.pdf (accessed 17 December 2024).
